# Global research trends in enteric nervous system and gut microbiota: a bibliometric analysis (2005-2025)

**DOI:** 10.3389/fcimb.2026.1806356

**Published:** 2026-08-26

**Authors:** Wang Tao, Yunfeng Yu, Xiangning Huang, Jiawang Huang, Neng Wang, Xueli Shangguan, Rong Yu, Fan Xiao

**Affiliations:** School of Traditional Chinese Medicine, Hunan University of Chinese Medicine, Changsha, Hunan, China

**Keywords:** bibliometric, citespace, enteric nervous system, gut microbiota, neuroinflammation, Parkinson’s disease, probiotic, VOSviewer

## Abstract

**Background:**

The crosstalk between the enteric nervous system (ENS) and gut microbiota, along with their roles in disease, has garnered increasing scientific attention. However, a comprehensive and objective analysis of the field’s current status and evolutionary trajectory remains lacking.

**Methods:**

We retrieved and screened relevant publications from the Web of Science Core Collection and Scopus database. Bibliometric and visual analysis were performed using CiteSpace, VOSviewer, R-studio, Origin and Excel 365.

**Results:**

A total of 759 relevant articles and reviews published from 2005 to 2025 were included. The annual publication volume exhibited a discernible three-phase growth trajectory, culminating in a peak output in recent years. Collaboration network analysis revealed the United States as the most influential country. Leading institutions, based on publication output and centrality metrics, included University College Cork (Ireland), McMaster University (Canada), the University of Melbourne (Australia) and Baylor College of Medicine (the US). Key journals publishing in this field included the *International Journal of Molecular Sciences* and *Neurogastroenterology and Motility*. Author cooperation analysis highlighted John F. Cryan (Ireland) as the most influential scholar, with his seminal review “The Microbiota-Gut-Brain Axis” being the highly cited reference. Furthermore, keywords and co-cited reference analysis revealed that current research primarily centers on specific disorders such as irritable bowel syndrome, inflammatory bowel disease, Parkinson’s disease and Alzheimer’s disease. The pivotal role of neuroinflammation and neuro-immune interactions was consistently identified as a core pathological mechanism. Importantly, modulation of the gut microbiota via probiotics and microbial metabolites like short-chain fatty acids emerged as predominant potential therapeutic targets.

**Conclusion:**

Research on the gut microbiota and the ENS elucidates the mechanisms and potential therapeutic directions for a spectrum of disorders, including irritable bowel syndrome, inflammatory bowel disease, Parkinson’s disease, and Alzheimer’s disease. Fostering international collaboration and interdisciplinary exchange is crucial to accelerate the translation of high-quality research into clinical applications.

## 1 Introduction

The enteric nervous system (ENS), often referred to as the “second brain of the gut”, constitutes a complex neuronal network in the gastrointestinal tract. Derived from vagal neural crest progenitors during embryonic development, the ENS comprises millions of neurons and glial cells that are organized into myenteric (Auerbach’s) and submucosal (Meissner’s) plexuses (Nguyen et al., 2023). The myenteric plexus extends along the entire digestive tract from the esophagus to the rectum, whereas the submucosal plexus, located in the submucosa of the small and large intestines (Pawolski and Schmidt, 2020). As early as 1755, Albert Von Haller observed that intestinal peristalsis could persist even after disconnection from the central nervous system (CNS), a finding that revealed for the first time the ENS’s ability to independently regulate gastrointestinal motility (Gershon, 1998). Structural and functional disruptions of the ENS are thus implicated in a range of gastrointestinal disorders, including irritable bowel syndrome (IBS) and inflammatory bowel disease (IBD) (Stavely et al., 2023). Furthermore, as a pivotal hub in the gut–brain axis, the ENS maintains close bidirectional communication with the CNS, and its dysfunction has been closely linked to the pathogenesis of neurodegenerative diseases such as Parkinson’s disease (PD) and Alzheimer’s disease (AD) (Fried et al., 2021).

In recent years, the interaction between the ENS and gut microbiota has gained an increasingly prominent research focus (Sharkey and Mawe, 2023). As the most complex microecosystem in the digestive tract, gut microbiota exerts a profound influence on the development, maturation, and functional integrity of the ENS. Studies have shown that maternal gut dysbiosis during pregnancy could impair the normal development process of the fetal ENS (Sajdel-Sulkowska, 2023). Furthermore, in antibiotic-induced germ-free animal models, adult mice exhibit a reduction in enteric neurons and glial cells, vagal afferent degeneration, and phenotypes including small intestinal lengthening and delayed gut transit (Hung et al., 2020; Vicentini et al., 2021). Notably, these deficits might be reversed by reestablishing microbial balance by interventions like short-chain fatty acid (SCFAs) supplementation or colonization with particular bacteria like *Bacteroides*, which would enhance gastrointestinal motility and restore cholinergic neuron activity (Aktar et al., 2020). These findings directly confirm the crucial role of gut microbiota in maintaining ENS homeostasis. Given that the ENS acts as a core hub of the gut-brain axis, further research indicates that the regulatory effect of the gut microbiota on the ENS may extend to the CNS. For instance, in animal models of PD, gut dysbiosis promote abnormal aggregation of α-synuclein (α-syn) in the ENS, trigger neuroinflammation, and potentially transmit these pathological effects to the brain via neural pathways such as the vagus nerve (Wang et al., 2021). Therefore, elucidating the interaction mechanism between the gut microbiota and the ENS may not only help clarify the pathophysiological basis of related disorders but also open up novel therapeutic avenues for gastrointestinal and neurodegenerative diseases.

Bibliometrics, first coined by Pritchard in 1969, is a research approach that applies mathematical and statistical methods to conduct quantitative and qualitative analysis of literature in a specific field over a specified time frame (Pritchard, 1969). By examining core elements including countries, institutions, journals, authors, keywords, and co-citations, this method can objectively reveal the developmental trajectories, collaborative networks, and research hotspots of a discipline (Wan et al., 2022). Although research on the ENS and gut microbiota has evolved significantly over the past two decades, a comprehensive bibliometric analysis in this area is still lacking. Given the ability of bibliometrics to integrate and visualize fragmented research, this study systematically analyzes relevant literature indexed in the Web of Science Core Collection (WoSCC) and Scopus database. In order to give researchers an evidence-based foundation for comprehending this domain’s evolutionary process and identifying new research directions, the goal is to map the research trends, knowledge structure, and frontier topics in this field.

## 2 Materials and methods

### 2.1 Literature source and search strategy

We collected the relevant research literature from the Web of Science Core Collection (WoSCC) and Scopus database. All searches were conducted on January 27, 2026 in both databases to avoid potential bias caused by database updates. WoSCC and Scopus are the predominant databases used in the medical field, renowned for their extensive disciplinary coverage, comprehensive citation indexing, and rich analytical metrics. These features enable researchers to accurately identify research hotspots and trends within a specific field. The combined use of these two databases ensures broad data sourcing for the bibliometric study, enhances the robustness of the research findings, and minimizes inherent database bias. We retrieved publications related to the “enteric nervous system” and “gut microbiota” from 2005 to 2025. Search syntax varies across databases, although the same core search terms were applied. The search terms were selected based on standardized terminology, PubMed MeSH Entry Terms, and relevant bibliometric studies to maximize retrieval sensitivity while maintaining consistency across databases. For instance, the search strategy used in the WoSCC is illustrated as follows: TS = [(Enteric Nervous system*) AND TS = (Gastrointestinal Microbiome* OR Microbiome, Gastrointestinal OR Microflora*, GI OR GI Microflora* OR Microbiome*, GI OR GI Microbiome* OR Enteric Microbiota* OR Microbiota*, Enteric OR Flora*, Enteric Microflora OR Enteric Microflora Flora* OR Microflora Flora*, Enteric OR Gut Microflora OR Microflora, Gut OR Gastrointestinal Microflora OR Microflora, Gastrointestinal OR Gastrointestinal Flora OR Flora, Gastrointestinal OR Gut Flora OR Flora, Gut OR Gastrointestinal Microbial Communit* OR Microbial Community, Gastrointestinal OR Gut Microbiome* OR Microbiome, Gut OR Gastrointestinal Microbiota* OR Microbiota, Gastrointestinal OR Microflora* OR Gut Microbiota* OR Microbiota, Gut OR Intestinal Microbiome* OR Microbiome, Intestinal OR Intestinal Microflora OR Microflora, Intestinal OR Intestinal Flora OR Flora, Intestinal OR Intestinal Microbiota* OR Microbiota, Intestinal OR Enteric Bacteria OR Bacteria, Enteric OR Gastric Microbiome* OR Microbiome, Gastric)].

### 2.2 Inclusion and exclusion criteria

To ensure the validity and reliability of the study, we first defined the eligibility criteria for literature selection. The inclusion criteria were as follows: (i) publications published between 2005 and 2025; (ii) English-language publications; (iii) peer-reviewed research articles or review articles; and (iv) studies addressing the interactions, mechanisms, regulation, or effects between the enteric nervous system (ENS) and the gut microbiota, or treating both as the central research focus.

The exclusion criteria were as follows: (i) duplicate records; (ii) retracted publications; (iii) non-peer-reviewed documents, such as conference abstracts, case reports, letters, editorials, and preprints; (iv) non-English publications; and (v) records unrelated to the ENS and the gut microbiota, including those referring only to the gut-brain axis or other nervous systems without explicitly involving the ENS, as well as studies that merely mentioned the relevant terms in the background without substantively discussing their relationship.

### 2.3 Data analysis and visualization

Literature retrieved from the WoSCC and Scopus databases was first merged and screened according to the predefined eligibility criteria. Duplicate records were removed in RStudio based on matching Digital Object Identifiers (DOIs), followed by secondary verification in CiteSpace to ensure record uniqueness. Subsequently, two researchers independently screened the records at the title, abstract, and full-text levels. Any disagreements were resolved through discussion, and a third reviewer was consulted when necessary. Before network construction and visualization, we standardized synonymous terms for countries/regions, institutions, authors, journals, and keywords to minimize inconsistencies across databases. After exporting the merged dataset, we generated map files in VOSviewer and manually reviewed the lists in Excel to identify variant spellings, abbreviations, and synonyms. We then applied the thesaurus.txt function in VOSviewer and the alias list function in CiteSpace to replace non-standard terms with standardized equivalents. The complete mapping list is provided in **Supplementary Material 2**.

To enhance the reliability and comprehensiveness of the bibliometric analysis, we used multiple complementary tools for cross-validation and visualization. VOSviewer (version 1.6.20), developed by Leiden University in the Netherlands, was used to visualize the distribution and collaboration of countries, institutions, authors, and keyword co-occurrence networks (van Eck and Waltman, 2010). Clustering was performed automatically based on similarity matrices and the VOS mapping technique, with cluster labels assigned by the researchers according to thematic content. CiteSpace (version 6.1.R2 Advanced), developed by Professor Chaomei Chen, was used to explore co-citation relationships, keyword timeline evolution, and citation bursts (Chen and Leydesdorff, 2014). In addition, Bibliometrix, ggplot2, and ComplexHeatmap in RStudio were used for data processing, statistical analysis, and supplementary visualizations such as heatmaps. Annual and cumulative publication trends were plotted using Microsoft Excel 365, and a chord diagram of international collaboration was generated with Origin 2024. We used multiple bibliometric indicators, including publication output, citation counts, total link strength, and H-index, to comprehensively assess the influence of countries/regions, institutions, authors, journals, and keywords. As all data were sourced from publicly available databases, no ethical approval was required for this study.

## 3 Results

### 3.1 Analysis of annual publications and future growth trends

**Figure 1:**
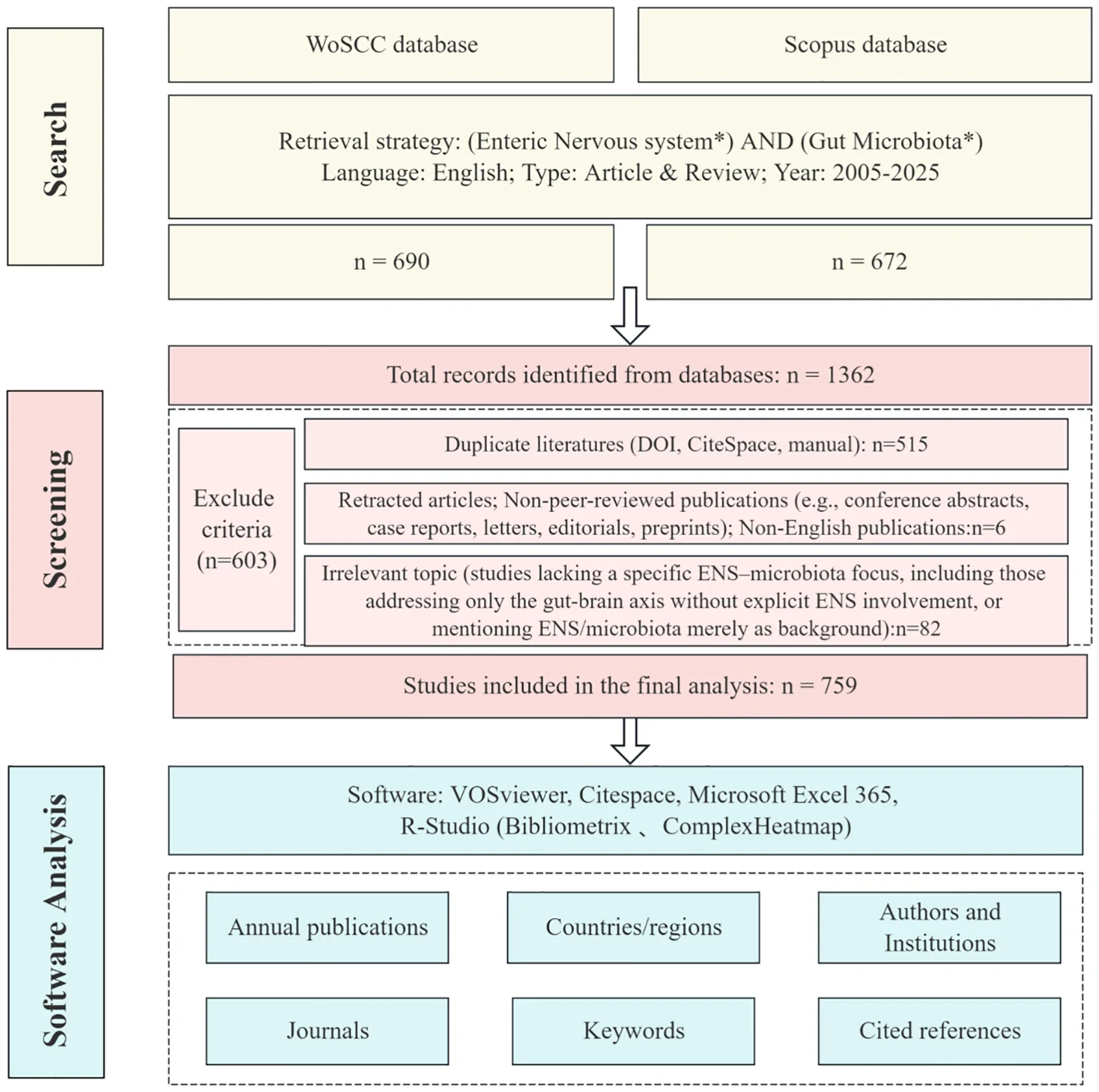
Flowchart of the literature screening process.

**Figure 2:**
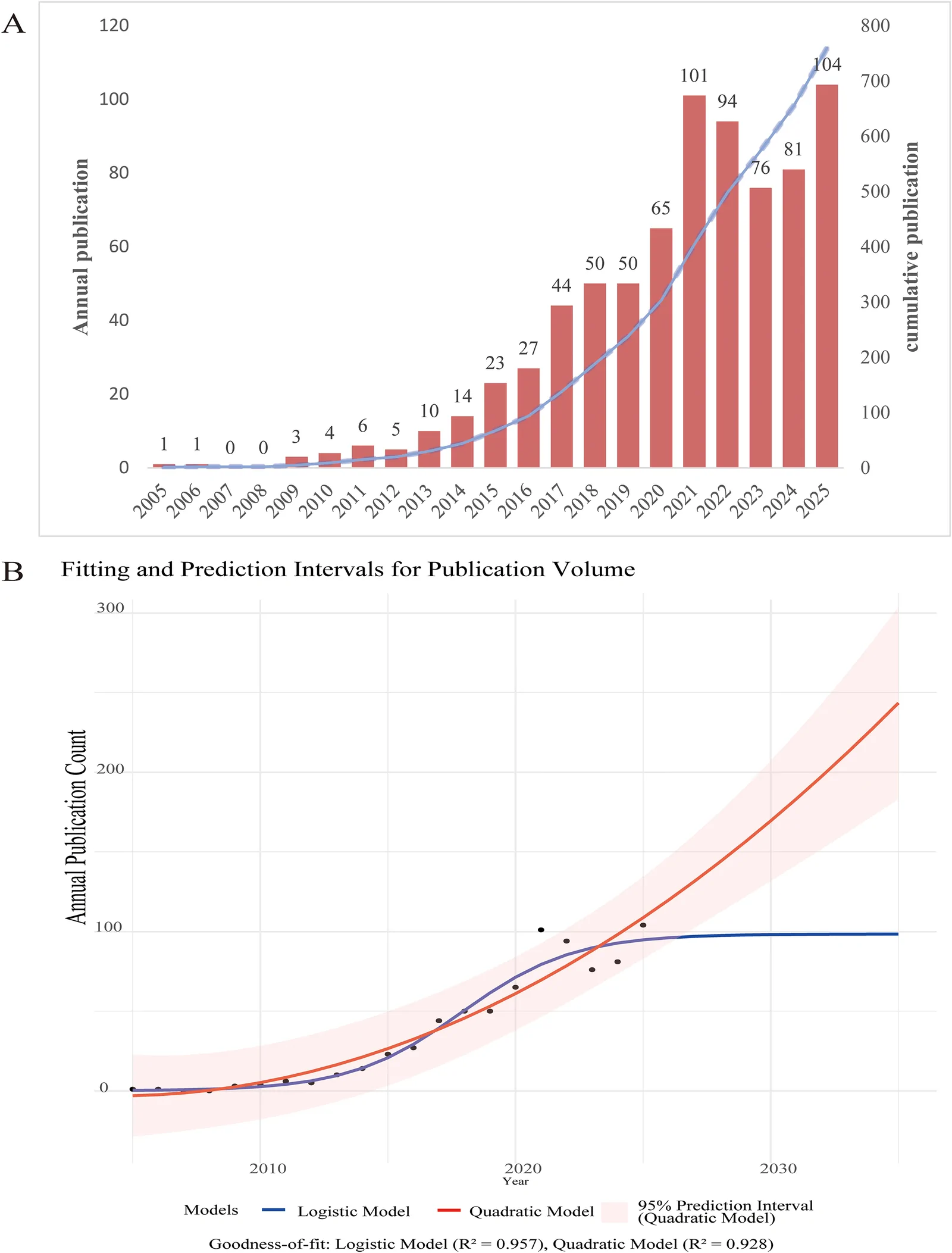
Annual publication volume and future forecasts **(A)** The number of related publications and cumulative publication for each year from 2005 to 2025. **(B)** Projections of annual publication output based on logistic growth and polynomial models.

A total of 759 articles were included in this study, including 378 articles and 381 reviews, as shown in **Figure 1**. The annual publication output exhibited a discernible three-phase trajectory (as shown in **Figure 2A**). The period from 2005 to 2010 was characterized by a low and slowly increasing volume. A phase of steady growth commenced in 2011 and escalated markedly between 2017 and 2021, culminating in a peak of 101 publications in 2021. Despite subsequent fluctuations, the annual output reached 104 by 2025, indicating that this field continues to attract sustained and vigorous scholarly attention. **Figure 2B** presents the logistic and quadratic polynomial models fitted to the annual publication volume. The scatter points represent the actual annual publication counts. The logistic growth model (blue curve) exhibits an excellent fit to the historical data (R² = 0.957), following a characteristic S-shaped pattern that predicts publication volume will approach saturation in the near future. In contrast, the quadratic polynomial model (red curve, R² = 0.928) exhibits a continuous upward trend. The shaded area around the red curve represents the 95% prediction interval for the quadratic polynomial model; this interval widens substantially during the forecast period (2025-2030), indicating considerable uncertainty in long-term predictions. Based on model comparison and prediction interval analysis, the logistic growth model better reflects the intrinsic growth pattern of this field. We anticipate that the annual publication output will stabilize at a high level while the growth rate is likely to stabilize.

### 3.2 Analysis of countries/regions

**Figure 3:**
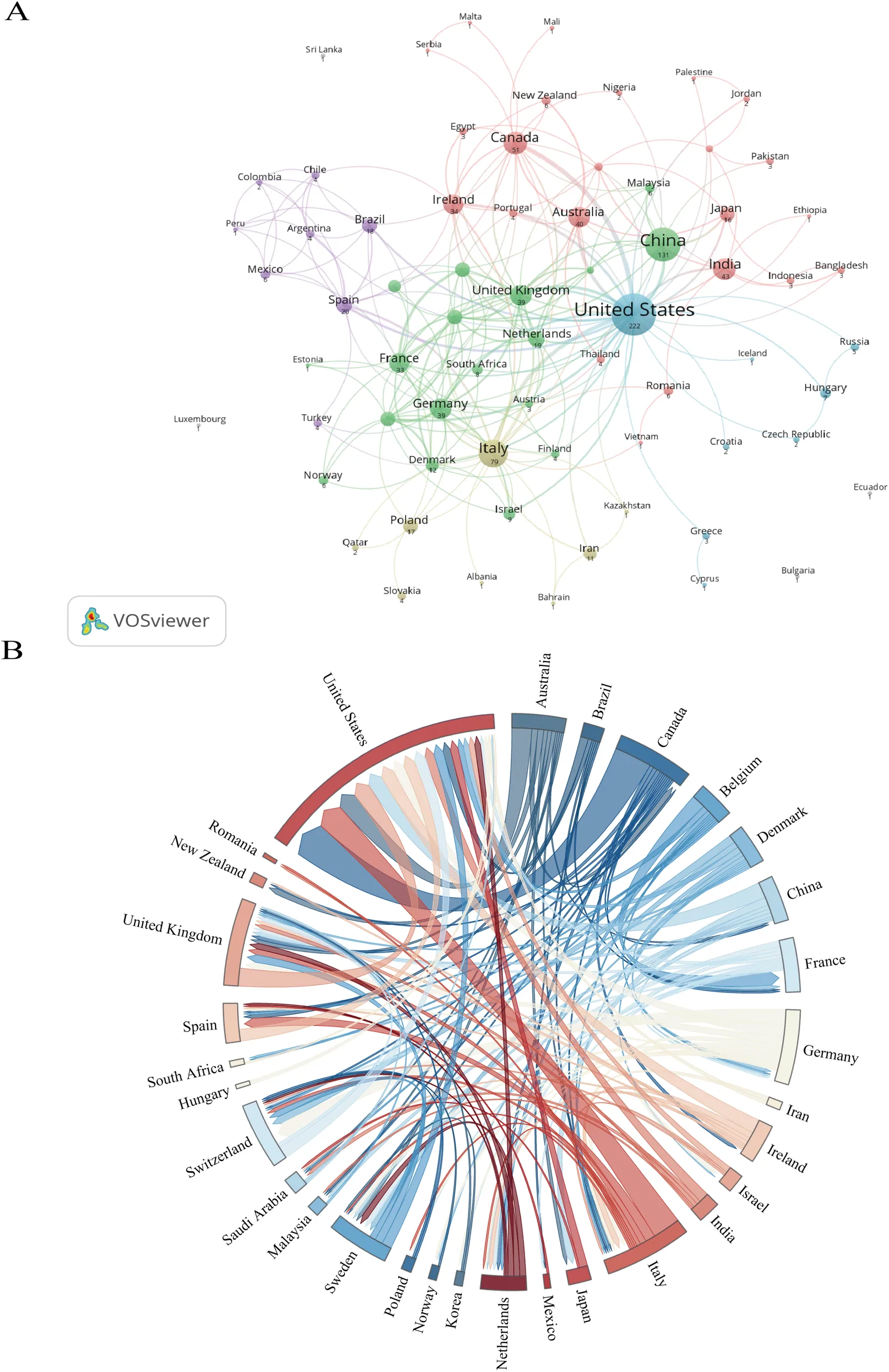
Distribution of country/region **(A)** Analysis of collaborative network visualization of countries/regions in VOSviewer. Node size represents publication output, and colors indicate different clusters. **(B)** Diagram of collaborative relationships between countries.

To date, 69 countries or regions worldwide have contributed to research on the ENS and gut microbiota. **Table 1** lists the top 10 countries/regions ranked by publication output, along with their corresponding citation counts and centrality values. In terms of publication volume, the United States (US) is the most productive country (222 articles), followed by China (119 articles) and Italy (69 articles). Also, the US leads in total citations (18066) and average citations per paper (81.38), consolidating its global leadership in this field. Notably, although Ireland and Canada have relatively modest publication outputs, they exhibit exceptionally high scientific impact, with average citations per paper of 296.79 and 150.53, respectively. Global collaboration network analysis performed with VOSviewer roughly grouped countries/regions into eight clusters based on collaboration intensity, each cluster represented by a distinct color (**Figure 3A**). The US serve as central research hubs in this field, maintaining extensive collaborative ties with multiple countries including Canada, Italy, Germany and the United Kingdom (**Figure 3B**). A national collaboration network visualized with CiteSpace (**Supplementary Figure 1**) further highlights the US’s exceptionally high level of international collaboration. The US (0.83), Italy (0.29) and Canada (0.22) and several other institutions show high centrality—an indicator reflecting a country’s pivotal role in connecting collaborative networks and integrating academic resources. Extensive international collaboration has effectively boosted the output of high-impact research, underscoring the bridging role of such collaboration in driving breakthrough progress. It is noteworthy that while China ranks second in publication output, its centrality remains low (0.05). This discrepancy may stem from the fact that Chinese collaborative research is concentrated among domestic institutions and insufficient cooperation with other international institutions. Furthermore, the red nodes for India and Ireland indicate high citation bursts, suggesting that research from these countries experienced a sharp increase in attention and citations during a specific period.

**Table 1:** Top 10 countries/regions in terms of number of publications, the corresponding frequency of citations and centrality.

Rank	Countries/regions	Publication (659, %)	Citations	Centrality	Citation per publication	Total link strength
1	United States	222 (29.25%)	18066	0.83	81.38	143
2	China	119 (17.26%)	4647	0.05	35.47	24
3	Italy	69 (10.41%)	6133	0.29	77.63	49
4	Canada	44 (6.72%)	7677	0.22	150.53	42
5	India	38 (5.67%)	1246	0.05	28.98	16
6	Australia	37 (5.27%)	2038	0.05	50.95	29
7	Germany	35 (5.14%)	3942	0.09	101.08	40
8	United Kingdom	35 (5.14%)	4182	0.11	107.23	47
9	Ireland	27 (4.48%)	10091	0.04	296.79	22
10	France	24 (4.35%)	2292	0.07	69.45	28

### 3.3 Analysis of institutions

A total of 1276 research institutions worldwide has contributed to research in this field. Publication output, citation counts, and centrality were used to characterize the productivity and influence of institutions (**Table 2**). In terms of publication volume, leading institutions are primarily from the US and Canada. University College Cork stands out for its exceptional academic influence, with 9912 total citations and an average of 330.40 citations per article. Baylor College of Medicine (0.17) and University College Cork (0.12) exhibit high centrality, reflecting its extensive international collaborations ties and pivotal role as an academic hub in this field (**Supplementary Figure 2**). Notably, McMaster University exhibited a high citation burst from 2012 to 2017, indicating that the institution produced a series of influential core publications that garnered significant scholarly attention during this interval.

**Table 2:** Top 10 institutions in terms of number of publications, citations and total link strength.

Rank	Institution	Publication (652, %)	Citations	Centrality	Citation per publication	Total link strength
1	University College Cork (Ireland)	30 (3.95%)	9912	0.12	330.40	24
2	McMaster University (Canada)	19 (2.50%)	4379	0.02	230.47	25
3	University of Melbourne (Australia)	16 (2.11%)	546	0.1	34.13	31
4	Baylor College of Medicine (the US)	13 (1.71%)	424	0.17	32.62	23
5	University of Padua (Italy)	13 (1.71%)	1209	0.01	93.00	20
6	University of Calgary (Canada)	12 (1.58%)	859	0.07	71.58	13
7	Harvard Medical School (the US)	10 (1.32%)	461	0.01	46.10	21
8	Michigan State University (the US)	10 (1.32%)	414	0	41.40	11
9	University of Applied Sciences Kaiserslautern (Germany)	10 (1.32%)	1258	0	125.80	19
10	University of Pisa (Italy)	9 (1.19%)	378	0.01	42.00	10

**Figure 4:**
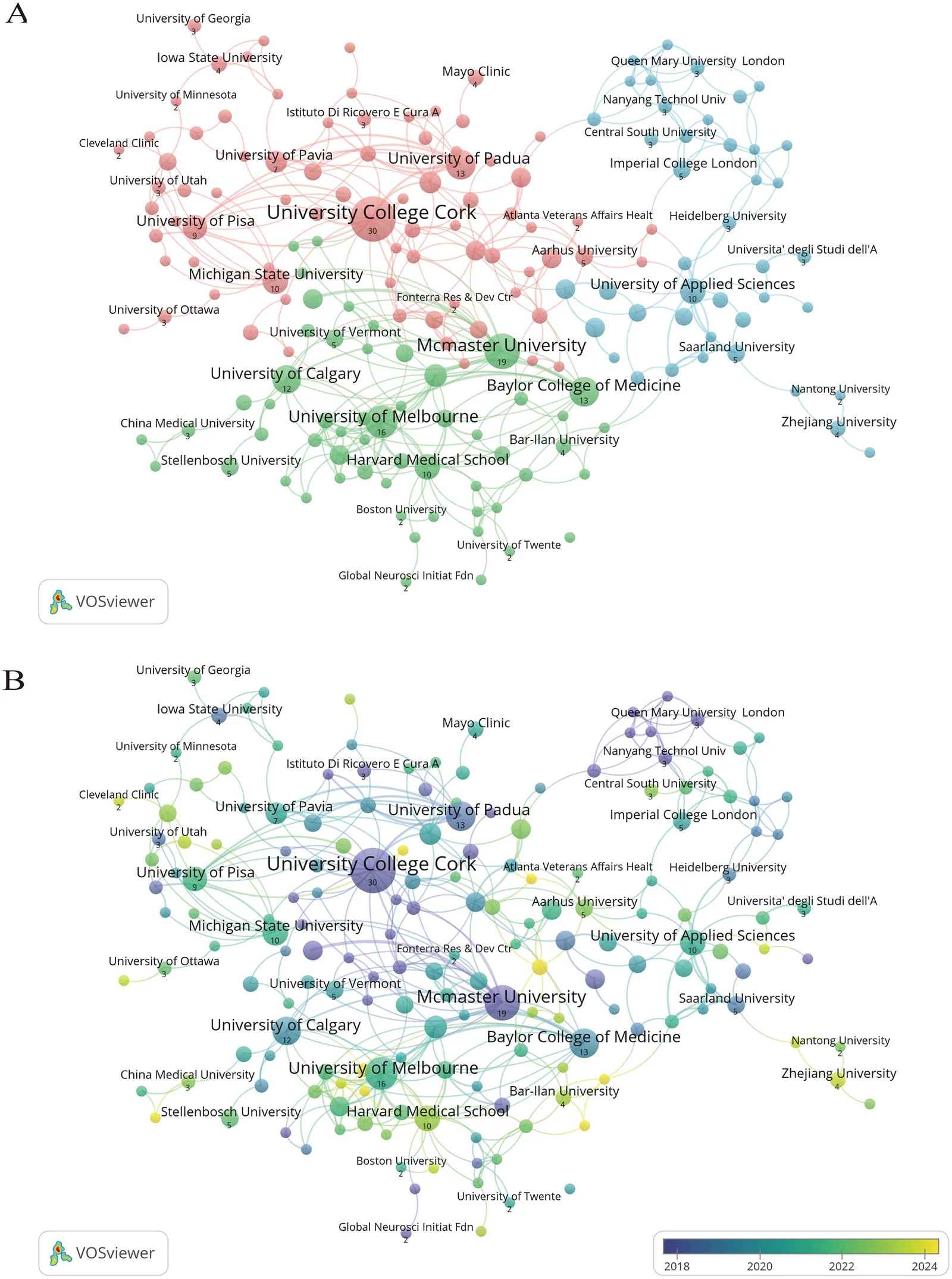
Analysis of related institutions **(A)** Visualization of collaboration network of institutions in VOSviewer, showing institutions with more than 2 publications. The nodes of different colors represent different clusters, and the size of nodes indicates frequency of occurrence. **(B)** Analysis of institutional activity based on publication output over the past five years. A yellow-shaded color indicates a higher proportion, suggesting that the institution is an emerging contributor with recent active engagement; a purple-shaded color represents a lower proportion, reflecting relatively decreased research activity in recent years.

We analyzed the institutional collaboration network using VOSviewer, which divided global institutions into three closely interconnected clusters (**Figure 4A**). These clusters include: a North America–Oceania cluster anchored by Baylor College of Medicine (the US), the University of Melbourne (Australia), McMaster University (Canada), and the University of Calgary (Canada); a Western European cluster centered around University College Cork (Ireland) and the University of Padua (Italy); and a Continental European cluster led by University of Applied Sciences Kaiserslautern (Germany). These cross-regional and inter-institutional collaborative networks effectively facilitate the dissemination of scientific findings via academic exchanges and resource sharing, thereby enhancing the overall quality and global visibility of research in this field. **Figure 4B** presents an analysis of institutional activity based on publication output over the past five years. This analysis identifies core institutions with currently active research contributions by calculating a standardized metric: the ratio of each institution’s publications from the recent five-year period to its total historical publication volume. The results show that institutions including Harvard Medical School (the US), Zhejiang University (China), Aarhus University (Denmark), and Bar-Ilan University (the State of Israel) have demonstrated a marked increase in research output, underscoring their significant role in shaping current research frontiers.

### 3.4 Analysis of authors

A total of 3,708 authors have contributed to this field. **Table 3** presents the most influential authors, comprehensively evaluated by publication output, citation counts, total link strength, and h-index. John F. Cryan (University College Cork, Ireland) ranks first in publication output (19), closely followed by Timothy G. Dinan (University College Cork, Ireland) (12) and Maria Cecilia Giron (University of Padua, Italy) (12). The most frequently co-cited author is also John F. Cryan (4249), followed by Timothy G. Dinan (2374) and Karl-Herbert Schaefer (University of Applied Sciences Kaiserslautern) (1495). It is worth noting that John F. Cryan exerts prominent influence in this field, as evidenced by both highly productive and the highest H-index (17) among all authors in this field. **Supplementary Figure 3** shows that Jaime PP. Foong and Wei Chen had high citation bursts in 2020-2025. This suggests that their work became a focal point in the field during this period and potentially helped to define or advance specific research directions.

**Table 3:** Top 10 authors in terms of number of publications, the corresponding institutions and total link strength.

Rank	Author	Publications	Institutions	H-index	Citations	Citation per publication	Total link strength
1	John F. Cryan	19	University College Cork (Ireland)	17	4249	223.63	35
2	Timothy G. Dinan	12	University College Cork (Ireland)	10	2374	197.83	27
3	Maria Cecilia Giron	12	University of Padova (Italy)	10	977	81.42	81
4	Valentina Caputi	10	University College Cork (Ireland)	9	878	87.8	63
5	Tor C. Savidge	10	Baylor College of Medicine (the US)	9	311	31.1	29
6	Karl-Herbert Schaefer	9	University of Applied Sciences Kaiserslautern (Germany)	8	1495	166.11	20
7	Joel C. Bornstein	8	University of Melbourne (Australia)	7	377	47.13	35
8	Cristina Giaroni	8	University of Insubria (Italy)	7	700	87.5	64
9	Claude Knauf	8	Institut de Recherche en Santé Digestive (France)	7	588	73.5	21
10	Keith A. Sharkey	8	University of Calgary (Canada)	8	645	80.63	18

**Figure 5:**
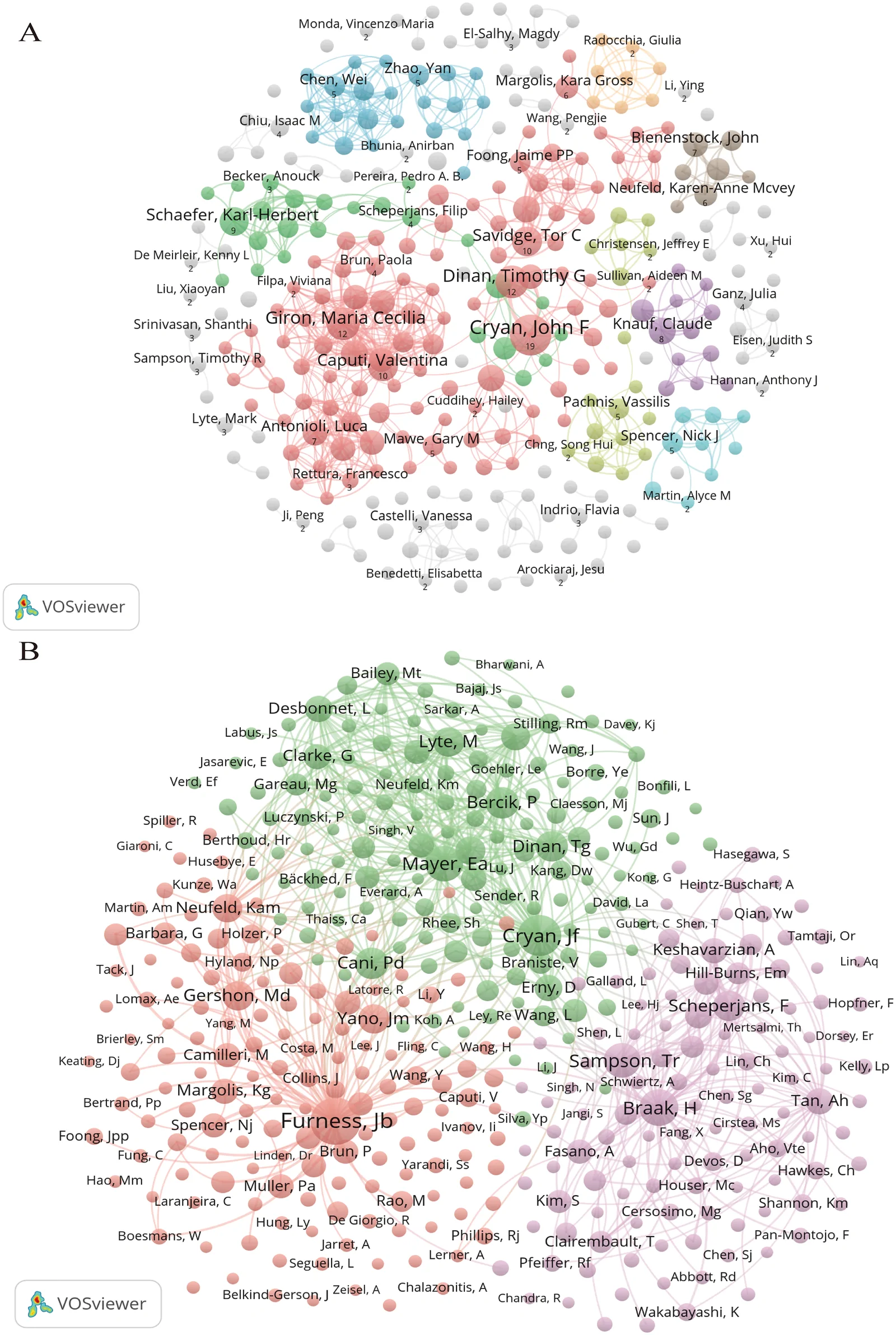
Analysis of related authors **(A)** Visualization of collaboration network of authors in VOSviewer, showing authors with more than 2 publications. **(B)** Visualization of co-citation network of authors in VOSviewer.

We also visualized the author collaborative network in VOSviewer (**Figure 5A**). It reveals that Maria Cecilia Giron, with the highest total link strength of 81 among all authors, maintains strong collaborative ties with Valentina Caputi, Cristina Giaroni, John F. Cryan, and Timothy G. Dinan. This core collaborative group bridges institutions across Ireland and Italy, highlighting her central role in the network. The author co-citation network (**Figure 5B**) further illuminates the highly focused research themes and their evolutionary relationships in this field. Authors are primarily clustered into three closely related groups: The red cluster, represented by foundational researchers like John B. Furness and Michael D. Gershon (hailed as the “father of neurogastroenterology”, who established the anatomical, developmental, neurochemical, and physiological foundations of the ENS), their work laid the theoretical groundwork for further research (Gershon and Tack, 2007); The green cluster, centered on John F. Cryan, Timothy G. Dinan, Emeran A. Mayer, and Patrice D. Cani, focuses on the “microbiota-gut-brain axis,” systematically exploring the effects of gut microbiota and their metabolites on the ENS, as well as their mechanistic roles in stress, anxiety, and cognitive behavior; The purple cluster, represented by Trisha L. Sampson and Heiko Braak, delves into the mechanisms of gastrointestinal dysfunction associated with neurodegenerative diseases like PD and AD.

### 3.5 Analysis of journals

Journal performance was examined in terms of publication output, citation frequency, impact factor (IF), and Journal Citation Reports (JCR) quartiles, as shown in **Table 4**. The journal with the highest number of publications was the *International Journal of Molecular Sciences* (4.9, Q1) (36), followed by *Neurogastroenterology and Motility* (3.3, Q2) (25), *Nutrients* (5.0, Q1) (17), and *Frontiers in Aging Neuroscience* (4.5, Q1) (13). Among the top 10 most productive journals, six are ranked in JCR Q1, and seven have an IF above 4. Notably, the most frequently co-cited journal was *Physiological Reviews* (28.7, Q1) (3565), followed by the Neurogastroenterology and Motility (2544) and *International Journal of Molecular Sciences* (2261). *Physiological Reviews* has only published 2 articles related to this topic, namely “The enteric nervous system” published by Keith A. Sharkey and “The Microbiota-Gut-Brain Axis” published by John F. Cryan. Both articles are landmark reviews that systematically synthesize foundational concepts and frontier progress in the field, explaining their high citation counts and long-term intellectual influence.

**Table 4:** Top 10 journals in terms of number of publications, corresponding IF (JCR 2024) and JCR quartile.

Rank	Journal	Publications	Citations	Total Link strength	Citation per publication	IF (JCR 2024)	JCR quartile
1	International Journal of Molecular Sciences	36	2261	287	62.81	4.9	Q1
2	Neurogastroenterology and Motility	25	2544	307	101.76	3.3	Q2
3	Nutrients	17	1078	168	63.41	5	Q1
4	Frontiers in Aging Neuroscience	13	569	119	43.77	4.5	Q1
5	American Journal of Physiology-Gastrointestinal and Liver Physiology	12	363	113	30.25	3.3	Q1
6	Frontiers in Neuroscience	12	443	94	36.92	3.2	Q2
7	World Journal of Gastroenterology	12	1108	66	92.33	5.4	Q1
8	Microorganisms	11	802	123	72.91	4.2	Q2
9	Cells	10	290	61	29.00	5.2	Q2
10	Frontiers in Microbiology	9	509	51	56.56	4.5	Q1

**Figure 6:**
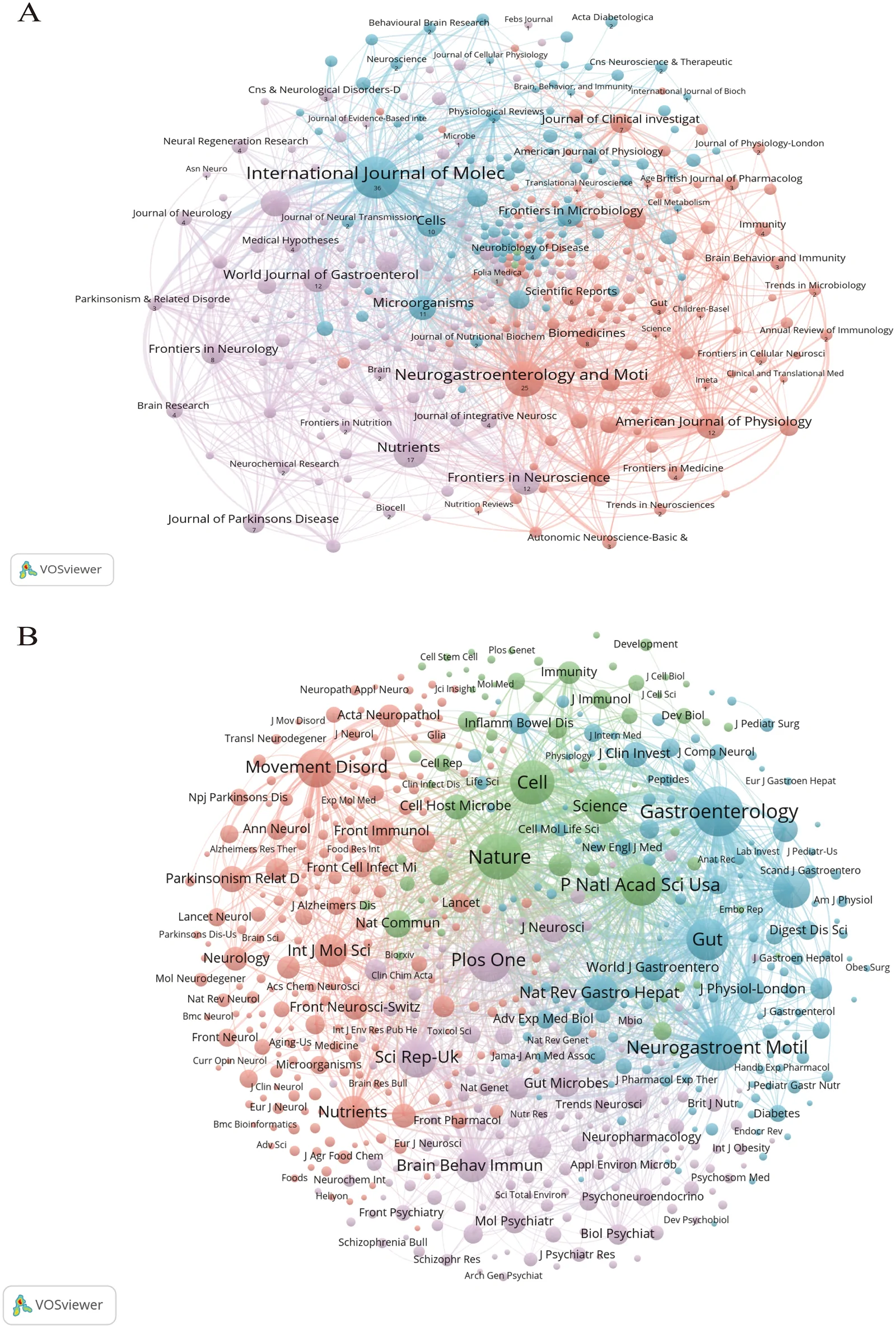
Analysis of related journals **(A)** Visualization of collaboration network of journals in VOSviewer, showing journals with more than 2 publications. Node size indicates frequency of occurrence, and colors represent different clusters. **(B)** Visualization of co-citation network of journals in VOSviewer.

**Figure 6A** presents the cooperative network structure of core journals through VOSviewer. The network shows that journals are closely connected, forming a highly interconnected research exchange circle. Based on journal similarity, the publications are broadly categorized into three clusters: The red cluster concentrates on gastrointestinal physiology and neuroimmune interactions, represented by journals such as *Neurogastroenterology and Motility*, *American Journal of Physiology-Gastrointestinal and Liver Physiology*, *Gastroenterology*, and *Brain, Behavior, and Immunity*. The blue cluster focuses on fundamental molecular cell biology and microbiological research, including journals like *International Journal of Molecular Sciences*, *Cells*, *Microorganisms*, and *Frontiers in Microbiology*. The purple cluster is centered on the pathology and clinical research of neurodegenerative diseases, with key journals being *Frontiers in Aging Neuroscience*, *Frontiers in Neurology*, and *Parkinsonism & Related Disorders*.

According to co-citation frequency, these journals can also be divided into four clusters (**Figure 6B**): the red cluster emphasizes neurological disorders such as Parkinson’s disease and molecular nutrition, featuring journals like *Movement Disorders*, *Nutrients*, *International Journal of Molecular Sciences*, and *Parkinsonism & Related Disorders*. The blue cluster is dedicated to gastroenterology and enteric nervous system research, represented by *Gut*, *Neurogastroenterology and Motility*, and *World Journal of Gastroenterology*. The green cluster encompasses fundamental immunology and high-impact, interdisciplinary science, including journals such as *Cell*, *Nature*, and *Science*. The purple cluster is core to gut microbiome research, with representative outlets being *Gut Microbes*.

### 3.6 Analysis of keywords

Keywords are standardized terms that reflect the core themes of publications, typically extracted from titles and abstracts to facilitate literature retrieval and thematic indexing. As a critical tool in bibliometric analysis, keywords enable the accurate identification of research frontiers, hotspots, and the knowledge structure of a discipline.

Beyond the original search terms, we analyzed author keywords extracted from the titles and abstracts of articles using VOSviewer. We identified a total of 4461 unique keywords, of which 186 appeared more than 10 times and 35 appeared more than 50 times. As shown in **Table 5**, disease-related keywords such as “Parkinson’s disease,” “irritable bowel syndrome,” and “inflammatory bowel disease” occurred frequently, this indicates that it is a common disease in this field of research. Additionally, keywords like “α-synuclein,” “gut-brain axis,” “inflammation,” “immune system,” “short-chain fatty acids,” and “probiotic” were also common, representing key pathological mechanisms and therapeutic targets in the field.

**Table 5:** Top 20 keywords in terms of frequency of occurrence and the corresponding total link strength.

Rank	Keyword	Occurrences	Total link strength
1	gut microbiota	486	4187
2	enteric nervous system	351	3041
3	parkinson disease	144	1443
4	chain fatty-acids	130	1276
5	gut-brain axis	116	1177
6	inflammation	93	861
7	α-synuclein	87	884
8	irritable bowel syndrome	82	769
9	brain	77	696
10	probiotic	77	781
11	immune system	67	622
12	mouse model	60	586
13	vagus nerve	50	529
14	dysbiosis	48	453
15	expression	48	432
16	inflammatory bowel disease	48	418
17	nervous system	48	476
18	anxiety	47	462
19	bacteria	46	443
20	neurons	45	408

**Figure 7:**
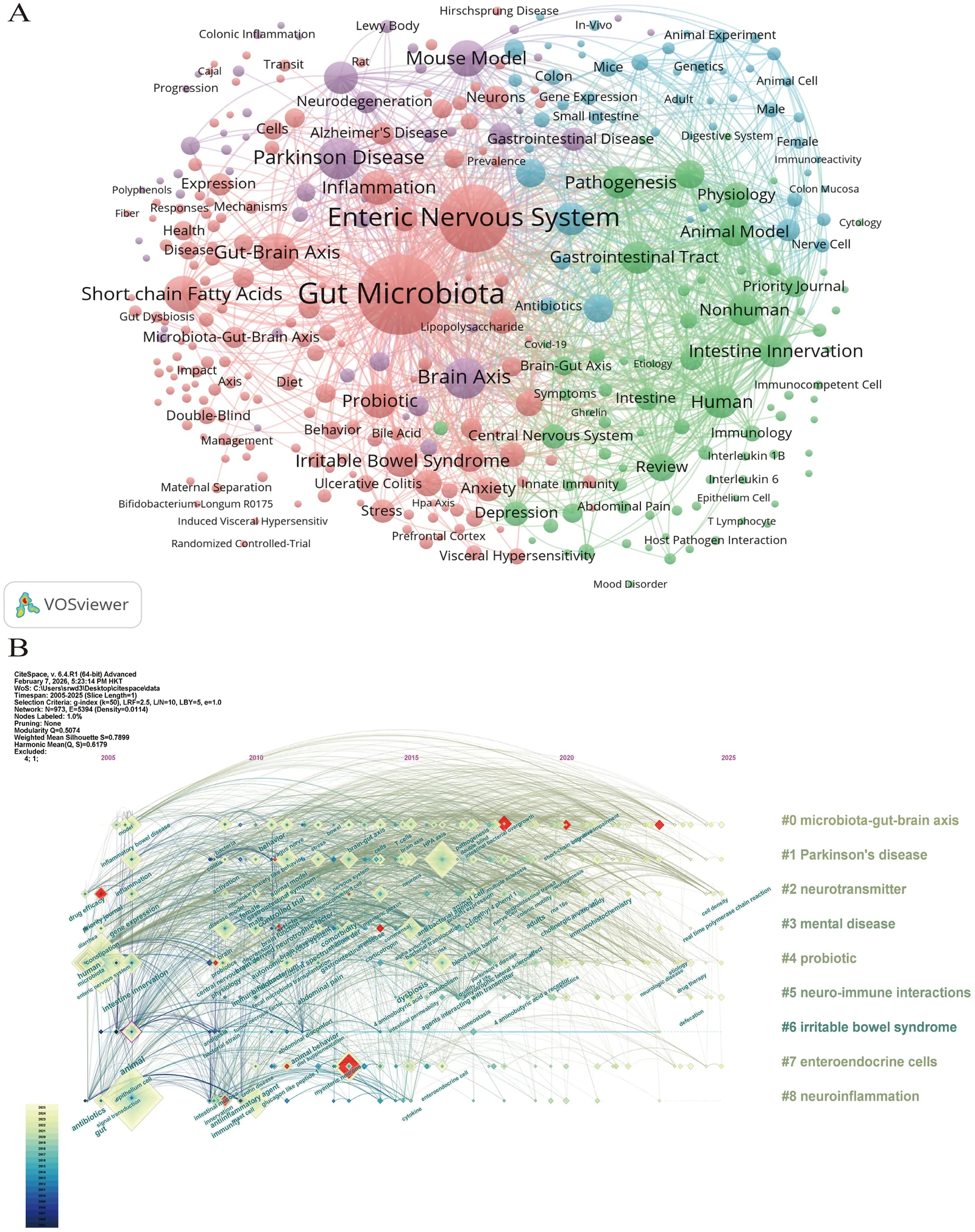
Analysis of keywords related to the ENS and gut microbiota **(A)** Keyword co-occurrence network (VOSviewer). This map displays keywords that appear more than 5 times. Node size represents frequency, and colors distinguish clusters. **(B)** Timeline view of keywords (Citespace). Each horizontal line represents a cluster. The smaller the number is, the larger the cluster, with #0 being the largest cluster. Nodes size reflects co-citation frequency, and the links between nodes indicate co-citation relationships. Nodes occurrence year is the time when they were first co-cited.

A co-occurrence network map of keywords was visualized in VOSviewer (**Figure 7A**). Based on research themes, the keywords were clustered into four primary groups: The light pink cluster centers on the interaction mechanisms between the gut microbiota and the ENS, encompassing themes such as inflammatory signaling, immune system, related disorders (e.g., irritable bowel syndrome, ulcerative colitis), and microbial therapeutics like probiotics. The light purple cluster is closely associated with the pathogenesis of neurodegenerative diseases, particularly Parkinson’s disease and Alzheimer’s disease, focusing on the gut-brain axis and the pathological transmission of proteins like α-synuclein. The light green and light blue clusters collectively address fundamental research methodologies and pathogenesis, emphasizing studies in human and animal models to elucidate disease mechanisms.

In CiteSpace, the timeline visualization displays the most prominent keywords within each cluster over time (**Figure 7B**). The earliest and largest cluster is #0 (microbiota-gut-brain axis). Within this cluster, recent research targets are “5-HT,” “toll like receptors” and “Alzheimer’s and Parkinson’s diseases”. Another early and sizable cluster is #1 (Parkinson’s disease), with research frontier including “permeability,” “*lactobacillus plantarum*,” “tryptophan metabolism,” and “butyrate.” Notably, six of the ten clusters remain academically active: #0 (microbiota-gut-brain axis), #2 (neurotransmitter), #4 (probiotic), #5 (neuro-immune interactions), #7 (enteroendocrine cells), and #8 (neuroinflammation), indicating that relevant research in this field is ongoing. Detailed information for **Figure 7B** is provided in **Supplementary Table 1**. **Supplementary Figure 4** identifies keywords with the strongest citation bursts during the 2023–2025 period: short-chain fatty acids, microbiota-gut-brain axis, neuroinflammation, health, and drug therapy. This pattern suggests that recent advancements in the field are closely associated with these research themes.

**Figure 8:**
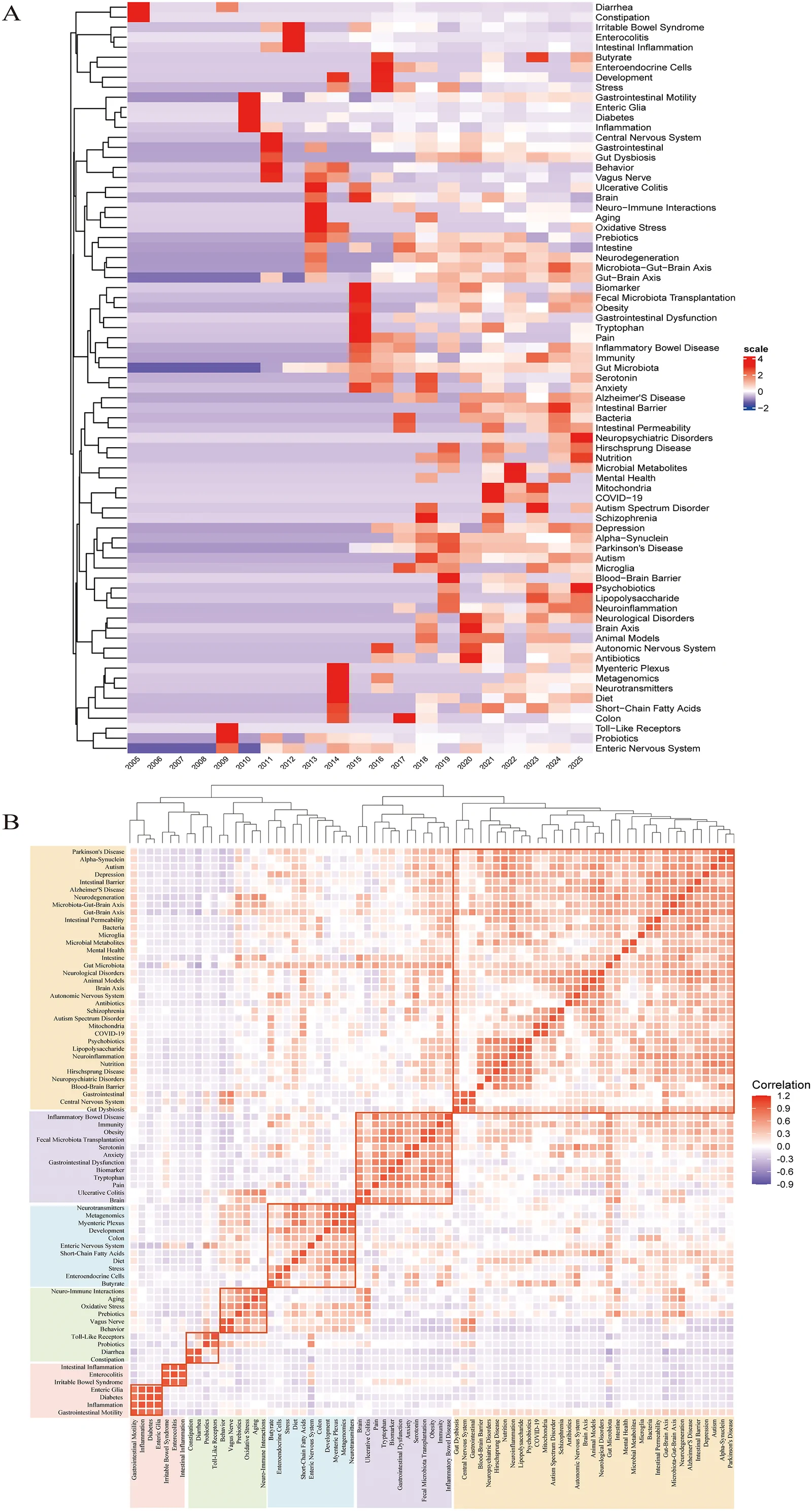
Heatmap analysis of keywords **(A)** Annual keyword heatmap from 2005 to 2025. The annual popularity score for each keyword is calculated as the ratio of its citations in a given year to its total citations. **(B)** Keyword correlation heatmap. Keywords with higher popularity within similar time periods are aggregated into clusters, each marked with a distinct color.

**Figure 8A** illustrates the annual popularity of keywords (defined as annual citation frequency relative to total annual citations). Terms like “intestinal permeability,” “neuroinflammation,” “immunity,” “inflammatory bowel disease,” “psychobiotics,” “intestinal barrier,” “lipopolysaccharide,” “Parkinson’s disease,” “Alzheimer’s disease,” “serotonin,” “butyrate,” and “microbiota-gut-brain axis” have achieved high recent popularity, indicating that these terms represent emerging research frontiers. **Figure 8B** displays the popularity correlations among keywords, with terms sharing similar popularity timelines clustered into five color-coded groups, each marked with a distinct color. The results reveal five major clusters: the pink cluster focuses on neuroinflammation and related disorders, featuring keywords such as “intestinal permeability,” “neuroinflammation,” “inflammatory bowel disease,” The green cluster is associated with intestinal immunity, covering terms like “neuro-immune interaction,” and “toll-like receptors.” The blue cluster centers on gastrointestinal motility disorders, including “neurotransmitters,” “enteroendocrine cells,” “myenteric plexus,” and “enteric nervous system.” The yellow cluster emphasizes gut-brain axis disorders, including terms like “microbiota-gut-brain axis,” “neurological disorders,” “Parkinson’s disease,” “Alzheimer’s disease,” “Hirschsprung’s disease,” “autism spectrum disorder,” “depression. “Keywords within the same cluster tend to have higher contemporaneous popularity and stronger interconnections.

### 3.7 Analysis of high cited references

The high co-citation frequency of a reference reflects its foundational value in a field (Small, 1973). We summarized the top 10 most frequently co-cited references in this field, including 7 original articles and 3 reviews (**Table 6**). The most highly co-cited reference is “Gut Microbiota Regulate Motor Deficits and Neuroinflammation in a Model of Parkinson’s Disease” by Sampson et al. (2016). This seminal work provided direct experimental evidence that the gut microbiota influences PD-related gastrointestinal motility dysfunction, specifically through mechanisms involving αSyn pathology in the ENS and microglial activation, positing that alterations in the microbiome constitute a risk factor for PD. The second most co-cited reference, “The Microbiota-Gut-Brain Axis” by Cryan, John F (2019) (Cryan et al., 2019), offers a systematic framework for the bidirectional communication between the gut microbiota and the brain, detailing mediating pathways including immune signaling, tryptophan metabolism, microbial metabolites, the vagus nerve, and the ENS. Following this, the work by Unger et al. (2016), “Short-chain fatty acids and gut microbiota differ between patients with Parkinson’s disease and age-matched controls” [20], demonstrated specific gut microbial shifts (e.g., decreased *Prevotellaceae*, increased *Enterobacteriaceae*) and reduced short-chain fatty acid (SCFA) levels in PD patients (Unger et al., 2016). It proposed that these SCFA reductions may induce changes in the ENS and contribute to gastrointestinal dysmotility in PD.

**Table 6:** Top 10 highly cited references.

Rank	Article title	Source title	Authors	Frequency	Year	DOI
1	Gut Microbiota Regulate Motor Deficits and Neuroinflammation in a Model of Parkinson’s Disease	Cell	Sampson et al. (2016)	72	2016	10.1016/j.cell.2016.11.018.
2	THE MICROBIOTA-GUT-BRAIN AXIS	Physiological Reviews	Cryan et al. (2019)	70	2019	10.1152/physrev.00018.2018
3	Short chain fatty acids and gut microbiota differ between patients with Parkinson’s disease and age-matched controls	Parkinsonism & Related Disorders	Unger et al. (2016)	54	2016	10.1016/j.parkreldis.2016.08.019
4	Parkinson’s disease and Parkinson’s disease medications have distinct signatures of the gut microbiome	Movement Disorders	Hill-burns et al. (2017)	46	2017	10.1002/mds.26942.
5	Neuronal programming by microbiota regulates intestinal physiology	*Nature*	Obata et al. (2020)	45	2020	10.1038/s41586-020-1975-8
6	Gut Microbiota Are Related to Parkinson’s Disease and Clinical Phenotype	Movement Disorders	Scheperjans et al. (2015)	45	2015	10.1002/mds.26069
7	Transneuronal Propagation of Pathologic α-Synuclein from the Gut to the Brain Models Parkinson’s Disease	*Neuron*	Kim et al. (2019)	44	2019	10.1016/j.neuron.2019.05.035
8	Indigenous bacteria from the gut microbiota regulate host serotonin biosynthesis	Cell	Yano et al. (2015)	43	2015	10.1016/j.cell.2015.02.047
9	The Role of Short-Chain Fatty Acids From Gut Microbiota in Gut-Brain Communication	Frontiers in Endocrinology	Silva et al. (2020)	39	2020	10.3389/fendo.2020.00025
10	Enteric nervous system: sensory transduction, neural circuits and gastrointestinal motility	Nature Reviews Gastroenterology & Hepatology	Spencer and Hu (2020)	38	2020	10.1038/s41575-020-0271-2.

**Figure 9:**
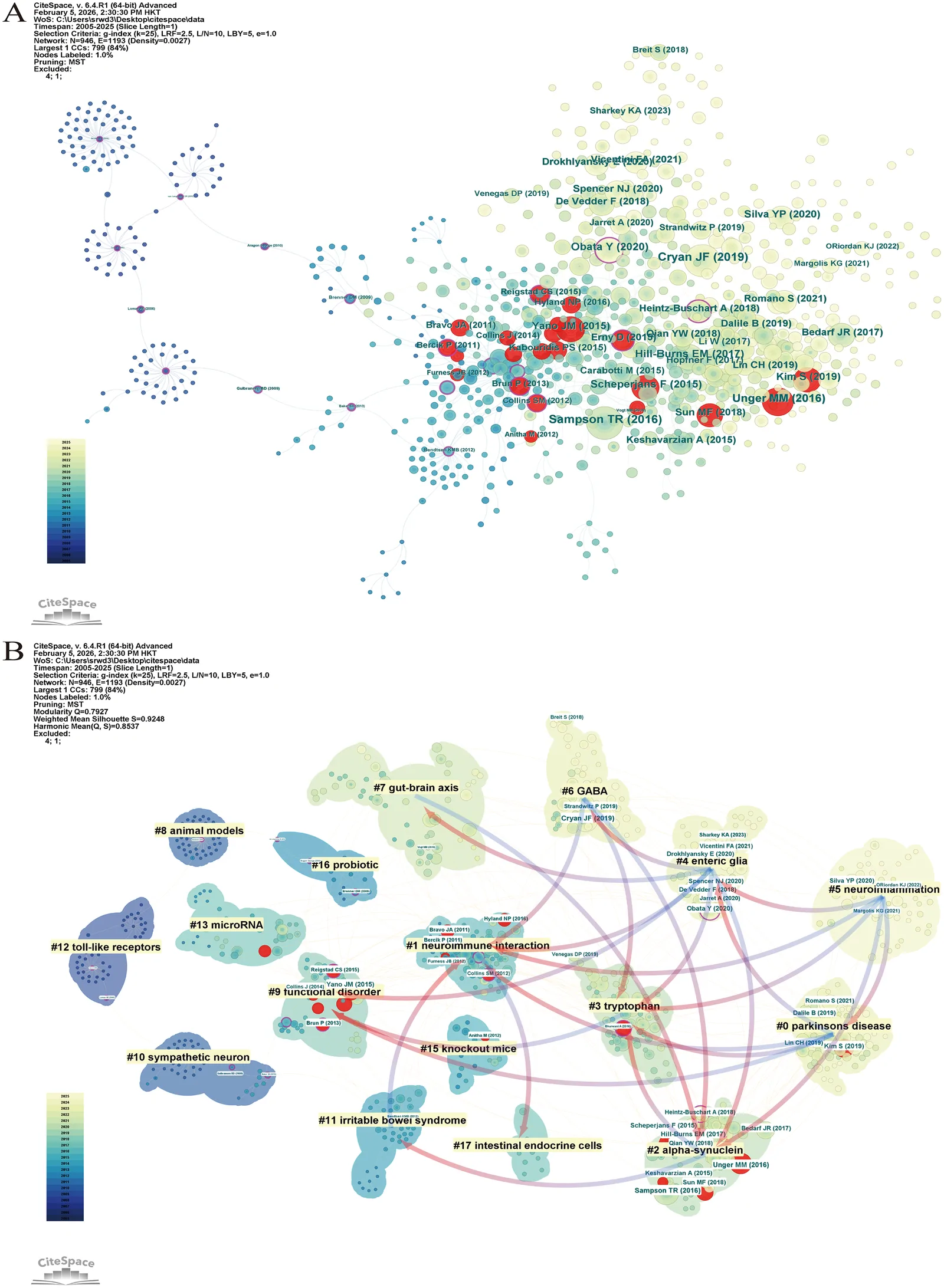
Analysis of cited references **(A)** Co-citation network of references (CiteSpace). Node size is proportional to the co-citation frequency. **(B)** Clustering of references based on their similarity, including #0 Parkinson’s disease, #1 Neuro-immune interactions, #2 alpha-synuclein, #3tryptophan, #4 enteric glia, #5 neuroinflammation, #6 GABA, #7 Gut-brain axis, #8 animal model, #9 functional disorder, #10 sympathetic neuron, #11 irritable bowel syndrome, #12 toll-like receptors, #13 microRNA, #15 knockout mice, #16 probiotic, #17 intestinal endocrine cells.

Furthermore, a knowledge graph was generated in CiteSpace based on the complete set of co-cited references (**Figures 9A, B**). **Figure 9B** visualizes 17 distinct, color-coded clusters, revealing the temporal evolution of research themes. The largest and most recent cluster is #0 “parkinson’s disease”. Other actively evolving clusters include #4 “enteric glia”, #5 “neuroinflammation”, and #6 “GABA”, indicating sustained research interest in these areas. Notably, several thematic clusters, including #0 “Parkinson’s disease”, #4 “enteric glia”, #5 “neuroinflammation”, #6 “GABA”, and #11 “irritable bowel syndrome”, show convergent pathways into the overarching cluster #1, labeled “neuro-immune interaction”. This convergence underscores “neuro-immune interaction” as a central and integrative frontier in current interdisciplinary research, effectively linking fundamental ENS physiology, microbial regulation, and clinical pathology. The strongest citation bursts were concentrated within clusters #1 “neuro-immune interactions”, #2 “alpha-synuclein”, and #9 “functional disorder”, corresponding to the latest mechanistic breakthroughs in the field.

**Figure 10:**
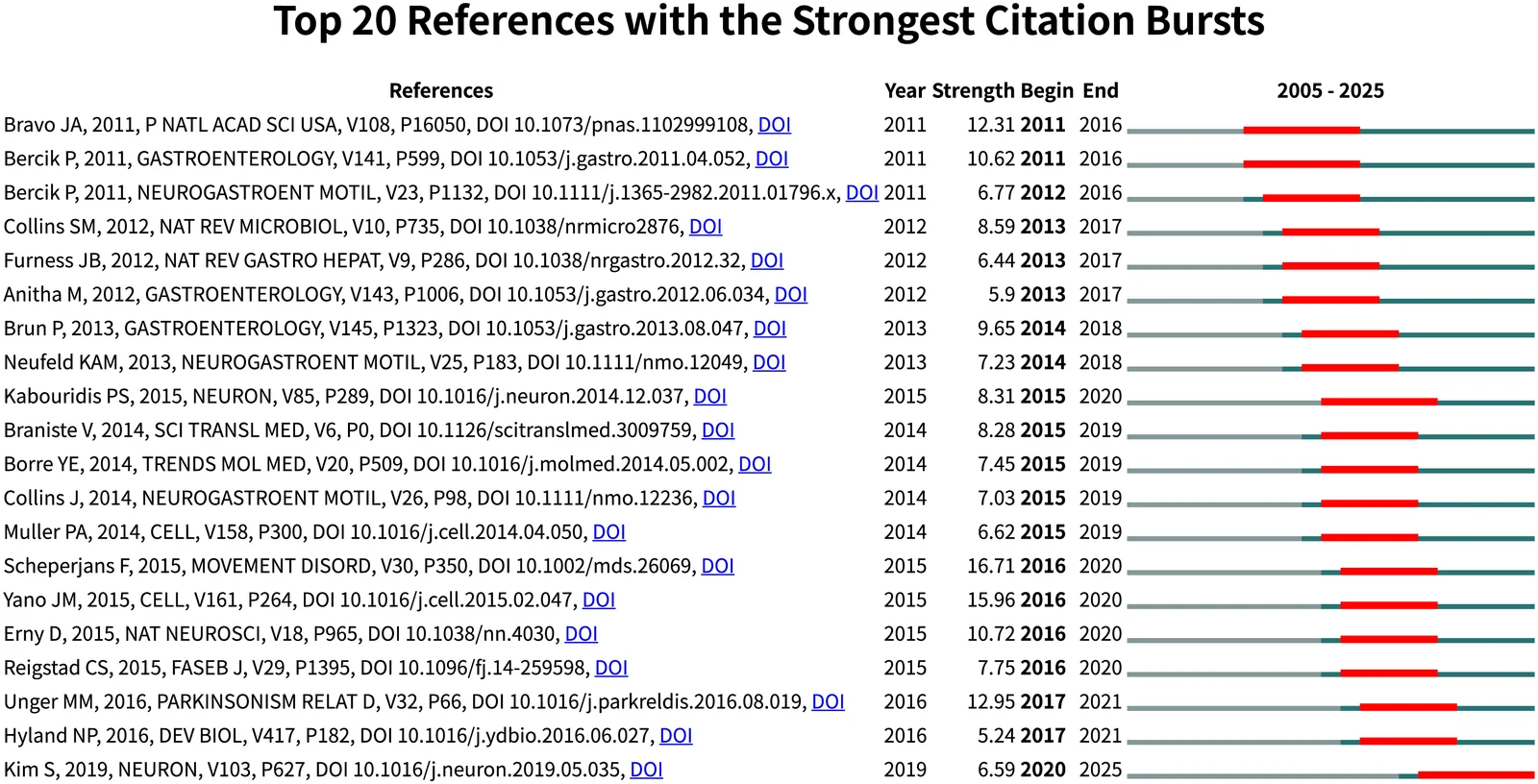
The top 20 references with the strongest citation bursts.

In addition, we identified the top 20 references with the strongest citation bursts (**Figure 10**). The earliest significant bursts appeared around 2011, associated with two landmark studies: “Ingestion of Lactobacillus strain regulates emotional behavior and central GABA receptor expression in a mouse via the vagus nerve” (Bravo et al., 2011) and “The Intestinal Microbiota Affect Central Levels of Brain-Derived Neurotropic Factor and Behavior in Mice” (Bercik et al., 2011). Notably, the reference with the strongest burst intensity (Strength = 16.71) was “Gut microbiota are related to Parkinson’s disease and clinical phenotype” by Scheperjans et al. (2015) (Scheperjans et al., 2015). This study suggests that α-syn-associated neurodegeneration in the ENS is linked to chronic constipation in PD, and reported a reduced abundance of *Prevotellaceae* and a positive correlation between *Enterobacteriaceae* abundance and the severity of postural instability/gait difficulty in PD patients, while acknowledging the need for further research to establish causality. Another highly burst reference was “Indigenous Bacteria from the Gut Microbiota Regulate Host Serotonin Biosynthesis” by Yano et al. (2015) (Strength = 15.96) (Yano et al., 2015), highlighting the critical role of gut microbiota and their metabolites in regulating intestinal and systemic serotonin (5-hydroxytryptamine, 5-HT) levels, with direct signaling implications for the ENS. Finally, the paper “Transneuronal Propagation of Pathologic α-Syn from the Gut to the Brain Models Parkinson’s Disease” by Kim et al. (2019) in *Neuron* (Kim et al., 2019), which showed a citation burst from 2020 to 2025, represents one of the most current research focal points.

## 4 Discussion

### 4.1 General trends

To systematically delineate the research landscape of the ENS and gut microbiota, this study employed bibliometric methods to analyze 759 relevant publications indexed in the WoSCC database from 2005 to 2025 based on bibliometric methods. Recent years have witnessed rapid growth in both annual publication output and total citations, underscoring that the ENS and gut microbiota, as an emerging interdisciplinary field, continue to attract increasing global scholarly attention. The widespread adoption of high-throughput 16S rRNA gene sequencing around 2013, enabling high-resolution identification and quantification of gut microbiota. This technological breakthrough directly drove a surge in research activity: publication output reached 101 articles in 2021, a tenfold increase compared to 2013 (10 articles), reflecting the field’s robust developmental trajectory. Turning to national and institutional contributions, the US, China, and Italy collectively account for over half of all publications, forming the core research cohort. The US led in terms of publication volume, citations, with Canada as its closest partner. Although Ireland and Canada had a moderate total output, they ranked first and second in average citations per article, respectively, reflecting high academic impact. Among research institutions, University College Cork (Ireland), McMaster University (Canada), and University of Melbourne (Australia), and stand out as the most prolific. Beyond sheer productivity, Baylor College of Medicine (the US) and University College Cork (Ireland) exhibited high centrality, demonstrating strong international collaborative capacity. Among authors, the most active contributors were John F. Cryan (Ireland), Timothy G. Dinan (Ireland) and Maria Cecilia Giron (Italy). The team led by Cryan, John F., focuses on the microbiota-gut-brain axis, systematically investigating how gut microbiota and their metabolites influence the ENS and their mechanistic roles in stress, anxiety, and cognitive behaviors (Aburto and Cryan, 2024; Gheorghe et al., 2024; O’Riordan et al., 2025).

### 4.2 Research hotspots and frontiers

Keyword analysis is an effective method to identify research frontiers and emerging trends in a specific field. By clustering high-frequency keywords, we found that current research predominantly revolves around terms such as “gut microbiota,” “enteric nervous system,” “neuroinflammation,” “immune system,” “gut-brain axis,” “Parkinson’s disease,” “irritable bowel syndrome,” “inflammatory bowel disease,” “short-chain fatty acids,” and “probiotics.” These keywords collectively outline three core knowledge modules in the field: mechanisms, associated diseases, and intervention strategies, forming a coherent research landscape that progresses from basic science toward clinical translation. The following sections will elaborate on these hotspots and the future trends they reflect.

#### 4.2.1 Mechanisms underlying gut microbiota-induced neuropathology

Current research on how gut microbiota drives neuropathology focuses on two interconnected hotspots: neuroinflammation and mucosal immunity. Neuroinflammation is a pivotal link bridging gastrointestinal disorders and neurodegenerative pathologies. Gut dysbiosis disrupts the balance between pro- and anti-inflammatory factors, triggering local neuroinflammation. This, in turn, damages enteric neurons, interferes with neurotransmitter release (e.g., 5-HT), and constitutes a central step in microbiota-related ENS dysfunction. Cattaneo et al (Cattaneo et al., 2017). showed that the gut of AD is enriched with pro-inflammatory bacteria (e.g., *Escherichia*/*Shigella*) and depleted of anti-inflammatory butyrate-producing bacteria. This dysbiosis correlates closely with increased cerebral amyloid β-protein (Aβ) plaque deposition and enhanced neuroinflammation. Studies by Keshavarzian (Keshavarzian et al., 2015) and Unger (Unger et al., 2016) revealed that patients with PD have increased abundances of *Enterobacteriaceae* and *Akkermansia*, and lower levels of *Prevotella*, *Roseburia*, *Blautia*, and *Faecalibacterium*. This shift increases intestinal permeability and reduces the level of SCFAs, fostering a state of “leaky gut.” The consequent translocation of lipopolysaccharide into the circulation allows it to bind to Toll-like receptor 4 (TLR4) on peripheral immune cells and brain microglia, promoting the release of pro-inflammatory cytokines such as tumor necrosis factor-alpha (TNF-α), interleukin-1β (IL-1β), and IL-6 (Caldarelli et al., 2024). These cytokines may not only contribute to degeneration of the ENS and α-syn pathology but can also impact the brain directly by crossing the compromised blood-brain barrier or indirectly via endothelial activation and signal transduction. This ultimately activates microglia, forming a core pathway that connects gut ecology to neurological diseases like PD and AD (Dogra et al., 2021).

The intestinal mucosal immune system, the first line of defense in host-microbiota interactions, has become another major focus in mechanistic research. It emphasizes the regulatory role of gut microbiota on immune development and function, primarily involving the differentiation of regulatory T cells and Th17 cells, as well as the role of secretory Immunoglobulin A in maintaining microbial homeostasis. SCFAs, beyond direct neurochemical modulation, could induce naive T cell differentiation into Tregs and other subsets, playing a crucial role in maintaining immune homeostasis and suppressing neuroinflammation (Jabbari Shiadeh et al., 2025). Additionally, intestinal epithelial and immunological cells are directly modulated by microbiota-derived ligands (such as TLRs and AhR ligands) and metabolites (like SCFAs), which impact systemic immune status (Gershon and Margolis, 2021). As key components of innate immunity, TLRs could recognize microbe-associated molecular patterns (Kabouridis and Pachnis, 2015). For instance, gut immune cascades are triggered when LPS from Gram-negative bacterial cell walls attaches to TLR4 on enterocytes (Mooser et al., 2025). Interestingly, TLR signaling also directly regulates the structure and function of the ENS: in adult mice, colonic microbiota can promote enteric neurogenesis and sustain nitrergic neuron activity through TLR2 signaling (Yarandi et al., 2020). Research by Anitha (Anitha et al., 2012) and (Yarandi et al., 2020) also found that TLR2 and TLR4 knockout mice develop intestinal motility disorders, accompanied by reduced inhibitory nitrergic neurons, a phenotype similar to that of germ-free mice. Moreover, excessive TLR activation triggers a cascade of pro-inflammatory cytokines, including interleukins and TNF-α, significantly contributing to neuronal dysfunction and cell death (de Araújo Boleti et al., 2025).

#### 4.2.2 Gut microbiota-associated neuropathology

Over the past two decades, gut microbiota has emerged as a central regulator of the gut-brain axis. Current research is increasingly elucidating bidirectional microbiota-brain communication via the ENS, vagus nerve, immune, and endocrine pathways. Particular attention is being paid to how this communication links gastrointestinal disorders to neurodegenerative diseases such as PD and AD.

The association between gut dysbiosis and gastrointestinal diseases like IBD and IBS is well-documented (Gu et al., 2019; Qiu et al., 2022). Dysbiosis is characterized by reduced beneficial bacteria (e.g., *Lactobacillus*, *Bifidobacterium*), enriched pathobionts (e.g., *Campylobacter*, *Clostridioides difficile*, *Escherichia coli*), and functional microbial alterations: for instance, patients with Crohn’s disease show a significant decrease in butyrate-producing *Faecalibacterium prausnitzii* alongside increases in *Actinomyces* and *Veillonella*; patients with ulcerative colitis have reduced *Eubacterium rectale* and *Akkermansia* alongside elevated *Escherichia coli* (Turpin et al., 2025); and IBS is associated with increased *Fusobacterium varium*, which correlates significantly with intestinal dysmotility and visceral hypersensitivity (Zheng et al., 2025). Such structural shifts in the microbiota lead to decreased production of beneficial metabolites (like SCFAs) and potentially increased levels of harmful metabolites (like trimethylamine N-oxide), collectively compromising intestinal mucosal barrier function. Barrier disruption enables antigen and endotoxin translocation, activating immune cells including mast cells, macrophages, and T lymphocytes, triggering a substantial release of pro-inflammatory cytokines (TNF-α, IFN-γ, IL-6) and establishing a state of systemic low-grade inflammation (Aziz et al., 2021). This chronic inflammation linked to the “leaky gut” is a major cause of central neuroinflammation, either through peripheral pathways like the vagus nerve or through damage to the blood-brain barrier.

Substantial evidence confirms that patients with IBD or IBS have a significantly higher risk of neurodegenerative diseases, including PD and dementia (Brudek, 2019; Liu et al., 2021; Zhang et al., 2021). In rat models of intestinal inflammation, pathological α-syn inclusions appear in the myenteric and submucosal plexuses of the ENS, accompanied by upregulated cerebral α-syn and substantia nigra dopaminergic neuron degeneration (Espinosa-Oliva et al., 2024). Furthermore, IBS-related intestinal permeability and chronic inflammation may promote gut β-amyloid formation, which may then influence the brain via neural pathways (Kuźniar et al., 2024). Below, we elaborate on microbiota crosstalk with typical gut-brain axis diseases (PD, AD), aligned with current research trends.

PD is the most representative gut-brain axis disease. According to the “Braak hypothesis,” pathological α-syn may originate in enteric neurons and retrogradely transmit to the brainstem via the vagus nerve, with gut dysbiosis considered a crucial “trigger” (Braak et al., 2003). Core pathological features of PD include dopaminergic neuron damage and abnormal α-syn aggregation. ENS-related gastrointestinal dysfunction (such as gastroparesis and constipation), often precedes typical motor symptoms. It may be an early pathogenic sign, as evidenced by the finding of α-syn aggregates in the ENS of prodromal PD patients (Stokholm et al., 2016).

Animal studies further confirm that ENS-derived α-syn aggregates retrogradely travel via the vagus nerve to the brainstem and substantia nigra, directly damaging dopaminergic neurons and exacerbating central neuroinflammation—a vicious cycle (Kim et al., 2019). With the application of 16S rRNA sequencing, researchers have found that the secretion of α-syn in the ENS is regulated by the gut microenvironment (Yuan et al., 2024). Consistent with highly cited studies, Scheperjans (Scheperjans et al., 2015) (Unger et al., 2016), et al. found increased *Enterobacteriaceae* in PD patients, which may impair the intestinal epithelial barrier and expose the ENS to luminal pathogens (Unger et al., 2016). further quantified reduced fecal SCFAs and their producers (e.g., *Faecalibacterium prausnitzii*) in PD patients; butyrate, which interacts with the ENS to enhance colonic contractions, may see its decline contributing to enteric neuropathy and gastrointestinal dysmotility (Yamada et al., 2015). The work of (Bedarf et al., 2017) provides additional evidence regarding metabolic pathways, noting decreases in *Prevotella* and SCFAs, an increase in *Akkermansia*, elevated β-glucuronidase activity, and altered tryptophan metabolism in PD patients. β-glucuronidase may weaken the gut barrier, increasing intestinal permeability. Pro-inflammatory dysbiosis can trigger gut-derived endotoxin responses, potentially leading to α-syn misfolding or neuronal damage. Forsyth et al (Forsyth et al., 2011). first linked intestinal permeability to PD onset, suggesting leaky gut may facilitate the translocation of *Escherichia coli* and endotoxins, inducing enteric neuron oxidative damage and abnormal α-syn accumulation. Liang et al (Liang et al., 2023). further demonstrated that *Escherichia coli* could induce colonic α-syn aggregation and transmit it to the CNS via the vagus nerve. Recently, Wu et al (Wu et al., 2025). revealed that *Dubosiella newyorkensis* might promote PD progression by interfering with branched-chain amino acid metabolism and inhibiting α-syn degradation. Collectively, these findings support a pathway whereby gut dysbiosis, via mechanisms like leaky gut and metabolic disturbances, induces inflammation and abnormal α-syn aggregation of the ENS. These pathological processes may then travel via the vagus nerve to the CNS, forming a key link between the gut and PD pathogenesis.

AD, another core neurodegenerative disease, is pathologically characterized by Aβ plaque deposition, hyperphosphorylated Tau neurofibrillary tangles, neuroinflammation, and neuronal death. Accumulating evidence indicates that gut dysbiosis, commonly observed in AD patients and models, may drive pathology by modulating peripheral or central Aβ deposition and Tau phosphorylation (Heneka et al., 2015). Harach et al (Harach et al., 2017). found reduced cerebral Aβ pathology in germ-free Amyloid Precursor Protein (APP) transgenic mice, while APP/PS1 mice showed decreased *Firmicutes*, increased *Bacteroidetes*, disrupted gut barrier homeostasis, and activation of innate immunity and glial cells, affecting central glial function via the ENS and blood-brain barrier. A clinical study by Grabrucker et al (Grabrucker et al., 2023). identified key bacterial genera and metabolites involved, noting that decreased abundance of *Clostridium* is associated with poor AD prognosis, *Coprococcus* may promote amyloid accumulation, and butyrate was identified as a key microbial metabolite negatively correlated with cortical amyloid load. According to Chen et al (Chen et al., 2022a), the absence of butyrate-producing anti-inflammatory bacteria in the guts of AD patients may result in immunological homeostasis and intestinal barrier disruption, microglial activation, and persistent neuroinflammation. An *in vitro* study confirmed that *Escherichia coli* endotoxin enhances Aβ fibril formation (Asti and Gioglio, 2014). In summary, gut dysbiosis, by affecting the ENS and altering gut-brain axis communication, constitutes a critical bridge from gut microenvironment disturbances to central Aβ and Tau pathology and neuroinflammation, though its precise mechanisms require further elucidation.

#### 4.2.3 Emerging therapeutic targets based on microbial modulation

Through metabolites like SCFAs, bile acids, and neuroactive chemicals like γ-aminobutyric acid (GABA) and 5-HT, the gut microbiota directly and indirectly affects the ENS, vagus nerve, and CNS. The two main approaches of current interventional research are direct microbial intervention (probiotics, prebiotics, fecal microbiota transplantation) or targeted modulation of key microbial metabolites (SCFAs, bile acids, tryptophan metabolism).

Evidence supporting the use of probiotics and prebiotics in gut-brain axis disorders is accumulating. Hall et al (Hall et al., 2023). found that a prebiotic mixture altered gut microbiota composition in PD patients, reducing pro-inflammatory *Proteobacteria* and improving gastrointestinal symptoms. In PD mouse models, *Lactobacillus* DP189 reshaped the gut microbiota by depleting pathogens and enriching beneficial bacteria, thereby decreasing α-syn aggregation in the ENS (Wang et al., 2022). *Pediococcus pentosaceus* alleviated dopaminergic neuron degeneration and α-syn pathology by modifying the microbiota and elevating intestinal GABA levels (Pan et al., 2022). Furthermore, an *in vitro* study by Harrass et al (Harrass et al., 2025). demonstrated that *Lactobacillus* probiotics could inhibit Aβ fibril formation and reduce Tau hyperphosphorylation and neuronal loss. These findings suggest that specific bacterial strains hold potential as targets for ameliorating gut-brain axis disorders, although their clinical efficacy and precise mechanisms of action require further validation through larger scale studies.

SCFAs, as highly scrutinized microbial metabolites, exert a complex “dual role” in the gut-brain axis. On one hand, SCFAs exert beneficial effects by activating G protein-coupled receptors or inhibiting histone deacetylases. These effects include anti-inflammatory actions, maintenance of intestinal barrier integrity, promotion of enteric neuron survival, modulation of α-syn aggregation, and alleviation of neuroinflammation and oxidative stress (Kalyanaraman et al., 2024). On the other hand, their mechanisms of action are closely tied to concentration, specific type, and host physiological status, leading to sometimes conflicting research conclusions. Wu et al (Wu et al., 2022). discovered that reduced serum propionate levels in PD patients were associated with motor symptoms, cognitive ability, and non-depressive status. In contrast, Chen et al (Chen et al., 2022b). reported decreased fecal SCFA levels but elevated plasma levels of specific SCFAs like propionate in PD patients, which might promote inflammatory responses. Additionally, animal research by Sampson et al. indicated that, at certain doses, SCFAs could even promote α-syn aggregation and exacerbate motor symptoms in a PD model (Sampson et al., 2016). These collective results underscore that the effects of SCFAs are highly dependent on their local microenvironment. Future research must delineate the specific conditions under which SCFAs exert beneficial versus detrimental effects.

Beyond SCFAs, the roles of tryptophan metabolites in regulating ENS and vagal nerve signaling are emerging as new research hotspots. For example, approximately 90% of the body’s 5-HT is produced in the gut by enterochromaffin cells, which convert dietary tryptophan via tryptophan hydroxylase 1. As a key neurotransmitter and paracrine signaling molecule, 5-HT regulates intestinal secretion, motility, and mucosal immune responses by acting on specific receptors located on enteric neurons, smooth muscle cells, and immune cells (De Vadder et al., 2018). Studies show that bacterial genera such as *Clostridium* and *Ruminococcus* can participate in tryptophan metabolism, influencing 5-HT synthesis, which in turn stimulates enteric neurons and modulates gut motility (Ye et al., 2021). This highlights the potential of targeting the tryptophan metabolism as a novel therapeutic strategy for managing gastrointestinal motility disorders associated with PD or AD.

### 4.3 Limitations and future prospects

This study is the first to systematically integrate VOSviewer and CiteSpace tools for bibliometric analysis of the ENS and gut microbiota, mapping the collaboration networks and key development trends. However, several limitations should be acknowledged. First, the literature was retrieved only from WoSCC and Scopus, which may have led to missing or duplicate records and limited database coverage. Second, the search strategy used “enteric nervous system” as the core term and did not explicitly include other ENS-related concepts, such as enteric neurons, enteric glia, and enteric plexuses, which may have excluded some relevant studies. Third, restricting the search to English-language publications may have introduced language bias. Fourth, the inclusion of both original articles and reviews may have affected citation-based analyses because of differences in citation patterns and knowledge contribution. Finally, citation-based indicators are time dependent; therefore, recently published high-quality studies may be underestimated, whereas older reviews may be overrepresented due to cumulative citation effects.

Future studies should expand database coverage, incorporate multilingual searches, and include additional ENS-related terms to more comprehensively capture the research landscape. Beyond that, future research should also advance the field in three key directions: 1) Deciphering the molecular mechanisms by which gut microbiota interacts with the ENS through pathways like immune regulation, neuroinflammation, and metabolite secretion, and how these interactions impact pathological processes like β-amyloid deposition or α-syn aggregation; 2) Determining and validating the particular microbial taxa or metabolites that are crucial to these pathologies, and evaluating their potential as intervention targets or diagnostic biomarkers; 3) Exploring the specific gut-brain axis targets of microbiota-based treatments (such as probiotics, prebiotics, and fecal microbiota transplantation) and expediting their translation into clinical applications.

## 5 Conclusion

Employing bibliometric methods, this study provides a systematic overview of the global research landscape and evolutionary trends in the field of the ENS and gut microbiota from 2005 to 2025. It offers a valuable macroscopic perspective for understanding the field’s development trajectory, research hotspots, and frontier directions. The field is currently in a phase of rapid expansion, with its intellectual structure primarily organized around core themes such as the gut-brain axis, neuroinflammation, mucosal immunity, and probiotics. Based on the present analysis, future research necessitates a deepened mechanistic exploration, precise identification of key targets, and a concerted push toward clinical translation. Strengthening interdisciplinary collaboration and incorporating innovative research methodologies hold significant promise for driving this field toward more profound and systematic development.

## Data Availability

The data presented in the study are deposited in the GitHub repository: https://github.com/taowang188/1806356.
