# Supplementary material for: Global research trends in enteric nervous system and gut microbiota: a bibliometric analysis (2005-2025)
Source: Front Cell Infect Microbiol. 2026 Aug 26;16:1806356. doi: 10.3389/fcimb.2026.1806356 (PMC13553251; doi:10.3389/fcimb.2026.1806356)
Supplement: Supplementary file 1 [file Table1.docx]

**1. Supplementary tables**

**Supplementary Table 1** Cooccurrence frequency, Burst, Degree, Centrality and Cluster_ID of the occurrence points in Figure 6B.

| Cluster ID | Keyword | Freq | Burst | Degree | Centrality |
| --- | --- | --- | --- | --- | --- |
| 0 | vagus nerve | 49 | 0 | 30 | 0.03 |
| 0 | vagotomy | 4 | 0 | 4 | 0 |
| 0 | tyrosine hydroxylase | 2 | 0 | 1 | 0 |
| 0 | toxicity | 2 | 0 | 3 | 0 |
| 0 | toll-like receptors | 2 | 0 | 6 | 0 |
| 0 | toll-like receptor 4 | 2 | 0 | 5 | 0 |
| 0 | T cells | 16 | 0 | 24 | 0.02 |
| 0 | system | 12 | 0 | 8 | 0 |
| 0 | symptoms | 5 | 0 | 1 | 0 |
| 0 | substantia nigra | 5 | 0 | 2 | 0 |
| 0 | stress | 37 | 0 | 20 | 0.01 |
| 0 | stimulation | 2 | 0 | 2 | 0 |
| 0 | short-chain fatty acids | 18 | 3.77 | 18 | 0.01 |
| 0 | sensory neurons | 10 | 0 | 13 | 0.01 |
| 0 | disease | 2 | 0 | 5 | 0 |
| 0 | responses | 6 | 0 | 4 | 0 |
| 0 | receptor expression | 3 | 0 | 3 | 0 |
| 0 | receptor | 39 | 0 | 21 | 0.01 |
| 0 | protein | 9 | 0 | 13 | 0 |
| 0 | progression | 2 | 0 | 3 | 0 |
| 0 | prefrontal cortex | 5 | 0 | 7 | 0 |
| 0 | plasticity | 2 | 0 | 3 | 0 |
| 0 | pathological alpha synuclein | 2 | 0 | 2 | 0 |
| 0 | pathogenesis | 22 | 0 | 30 | 0.03 |
| 0 | nonmotor symptoms | 11 | 0 | 10 | 0 |
| 0 | NF-κB | 4 | 0 | 6 | 0.01 |
| 0 | neuropsychiatric disorders | 4 | 0 | 2 | 0 |
| 0 | neuroinflammation | 9 | 3.53 | 15 | 0.01 |
| 0 | neuroimmune interactions | 2 | 0 | 2 | 0 |
| 0 | neuroendocrine system | 2 | 0 | 5 | 0 |
| 0 | neurodegenerative disease | 2 | 0 | 3 | 0 |
| 0 | neuro immune interactions | 4 | 0 | 7 | 0 |
| 0 | muscularis macrophages | 9 | 0 | 5 | 0 |
| 0 | motor deficits | 5 | 0 | 2 | 0 |
| 0 | motility | 16 | 0 | 7 | 0 |
| 0 | model | 41 | 0 | 17 | 0.01 |
| 0 | mitochondrial dysfunction | 2 | 0 | 1 | 0 |
| 0 | microbiota-gut-brain axis | 40 | 3.64 | 8 | 0 |
| 0 | metabolites | 2 | 0 | 2 | 0 |
| 0 | mechanisms | 13 | 0 | 13 | 0.02 |
| 0 | major depression | 15 | 0 | 15 | 0.01 |
| 0 | lipopolysaccharide | 4 | 0 | 9 | 0 |
| 0 | lewy body | 13 | 0 | 14 | 0.01 |
| 0 | kynurenine pathway | 2 | 0 | 10 | 0 |
| 0 | intestinal bacterial overgrowth | 18 | 0 | 17 | 0.01 |
| 0 | insights | 2 | 0 | 3 | 0 |
| 0 | innate lymphoid cells | 11 | 0 | 7 | 0 |
| 0 | injury | 2 | 0 | 5 | 0 |
| 0 | inflammatory bowel disease | 63 | 0 | 26 | 0.03 |
| 0 | in vitro | 12 | 0 | 16 | 0.02 |
| 0 | impact | 15 | 0 | 9 | 0 |
| 0 | immune system | 62 | 0 | 16 | 0 |
| 0 | immune cells | 2 | 0 | 1 | 0 |
| 0 | hydrogen sulfide | 4 | 0 | 1 | 0 |
| 0 | HPA axis | 7 | 0 | 26 | 0.02 |
| 0 | hirschsprune disease | 1 | 0 | 1 | 0 |
| 0 | gut inflammation | 13 | 0 | 12 | 0.01 |
| 0 | germ free mice | 2 | 0 | 2 | 0 |
| 0 | functional gastrointestinal disorders | 13 | 0 | 8 | 0 |
| 0 | epithelial barrier | 7 | 0 | 6 | 0 |
| 0 | endorectal pull through | 1 | 0 | 1 | 0 |
| 0 | dysfunction | 13 | 0 | 4 | 0 |
| 0 | downs syndrome | 1 | 0 | 1 | 0 |
| 0 | double blind | 26 | 0 | 19 | 0.01 |
| 0 | disorders | 28 | 0 | 15 | 0.01 |
| 0 | dietary polyphenols | 2 | 0 | 2 | 0 |
| 0 | dementia with lewy bodies | 2 | 0 | 2 | 0 |
| 0 | curcumin | 2 | 0 | 2 | 0 |
| 0 | commensal microbiota | 1 | 0 | 2 | 0 |
| 0 | colonization | 1 | 0 | 1 | 0 |
| 0 | colonic inflammation | 5 | 0 | 8 | 0 |
| 0 | cognitive impairment | 8 | 0 | 19 | 0.02 |
| 0 | clostridium difficile | 6 | 0 | 6 | 0.02 |
| 0 | clinical risk factors | 1 | 0 | 1 | 0 |
| 0 | cholinergic antiinflammatory pathway | 1 | 0 | 2 | 0 |
| 0 | cerebral ischemia/reperfusion injury | 2 | 0 | 2 | 0 |
| 0 | cells | 39 | 0 | 32 | 0.03 |
| 0 | butyrate | 6 | 0 | 15 | 0 |
| 0 | brain-gut axis | 16 | 0 | 35 | 0.03 |
| 0 | brain axis | 33 | 0 | 21 | 0.01 |
| 0 | bowel | 5 | 0 | 17 | 0.01 |
| 0 | bovine colostrum | 1 | 0 | 8 | 0 |
| 0 | bound phenolics | 1 | 0 | 2 | 0 |
| 0 | blood mononuclear cells | 1 | 0 | 2 | 0 |
| 0 | blood biomarkers | 1 | 0 | 5 | 0 |
| 0 | biogenic amines | 1 | 0 | 3 | 0 |
| 0 | bioaccumulation | 1 | 0 | 2 | 0 |
| 0 | bile salt hydrolase | 1 | 0 | 4 | 0 |
| 0 | benzalkonium chloride | 1 | 0 | 5 | 0 |
| 0 | behaviors | 1 | 0 | 3 | 0 |
| 0 | behavior | 29 | 0 | 47 | 0.04 |
| 0 | basal ganglia | 2 | 0 | 5 | 0 |
| 0 | barrier receptors | 1 | 0 | 5 | 0 |
| 0 | barrier integrity | 14 | 0 | 15 | 0.01 |
| 0 | barretts | 1 | 0 | 5 | 0 |
| 0 | bacterial outer membrane vesicles | 1 | 0 | 5 | 0 |
| 0 | bacteria | 45 | 0 | 32 | 0.03 |
| 0 | bacillus species | 1 | 0 | 1 | 0 |
| 0 | b cell responses | 1 | 0 | 1 | 0 |
| 0 | axis | 13 | 0 | 10 | 0 |
| 0 | autophagy | 2 | 0 | 8 | 0 |
| 0 | autonomic nervous system dysfunction | 1 | 0 | 3 | 0 |
| 0 | association | 10 | 0 | 8 | 0 |
| 0 | aspartate nmda receptors | 1 | 0 | 4 | 0 |
| 0 | appendix | 2 | 0 | 9 | 0 |
| 0 | apoptosis | 2 | 0 | 8 | 0 |
| 0 | anxiolytic effect | 1 | 0 | 8 | 0 |
| 0 | antineutrophil cytoplasmic antibody | 1 | 0 | 5 | 0 |
| 0 | antimicrobial peptide | 1 | 0 | 5 | 0 |
| 0 | antiinflammatory pathway | 1 | 0 | 4 | 0 |
| 0 | anti saccharomyces cerevisiae | 1 | 0 | 5 | 0 |
| 0 | anorexia nervosa | 1 | 0 | 3 | 0 |
| 0 | anorectal function | 2 | 0 | 9 | 0 |
| 0 | amines | 1 | 0 | 4 | 0 |
| 0 | alzheimers and parkinsons diseases | 1 | 0 | 7 | 0 |
| 0 | alzheimers | 1 | 0 | 5 | 0 |
| 0 | alpha-synuclein fibrils | 1 | 0 | 5 | 0 |
| 0 | alpha-synuclein (alpha-syn) | 1 | 0 | 5 | 0 |
| 0 | alpha synuclein expression | 3 | 0 | 13 | 0.01 |
| 0 | alpha synuclein accumulation | 1 | 0 | 5 | 0 |
| 0 | alpha defensins | 1 | 0 | 1 | 0 |
| 0 | alpha | 2 | 0 | 5 | 0 |
| 0 | all-trans retinoic acid | 1 | 0 | 1 | 0 |
| 0 | allosteric modulator | 2 | 0 | 3 | 0 |
| 0 | alcohol use disorder (aud)s | 1 | 0 | 8 | 0 |
| 0 | akkermansia muciniphila | 3 | 0 | 5 | 0 |
| 0 | agonist withdrawal syndrome | 1 | 0 | 3 | 0 |
| 0 | aggression | 1 | 0 | 5 | 0 |
| 0 | aggregation | 3 | 0 | 11 | 0.01 |
| 0 | affective science | 1 | 0 | 5 | 0 |
| 0 | adhesion | 1 | 0 | 1 | 0 |
| 0 | acylated ghrelin levels | 1 | 0 | 2 | 0 |
| 0 | acute psychological stress | 1 | 0 | 1 | 0 |
| 0 | acupuncture | 1 | 0 | 2 | 0 |
| 0 | acid binding proteins | 1 | 0 | 4 | 0 |
| 0 | acid | 5 | 0 | 13 | 0.01 |
| 0 | ace-2 receptor | 1 | 0 | 5 | 0 |
| 0 | 16s rrna pyrosequencing | 1 | 0 | 3 | 0 |
| 1 | type 1 diabetes | 2 | 0 | 5 | 0 |
| 1 | tryptophan metabolism | 17 | 0 | 9 | 0 |
| 1 | transit | 7 | 0 | 3 | 0 |
| 1 | tight junctions | 2 | 0 | 4 | 0 |
| 1 | synaptic plasticity | 2 | 0 | 3 | 0 |
| 1 | stem cells | 6 | 0 | 3 | 0 |
| 1 | smooth muscle | 14 | 0 | 7 | 0 |
| 1 | slow transit constipation | 2 | 0 | 3 | 0 |
| 1 | secretion | 2 | 0 | 1 | 0 |
| 1 | reuptake transporter | 2 | 0 | 5 | 0 |
| 1 | psychological stress | 2 | 0 | 4 | 0 |
| 1 | probiotic treatment | 2 | 0 | 3 | 0 |
| 1 | postnatal development | 9 | 0 | 3 | 0 |
| 1 | permeability | 6 | 0 | 2 | 0 |
| 1 | parkinson’s disease | 165 | 0 | 7 | 0 |
| 1 | neuroprotection | 2 | 0 | 2 | 0 |
| 1 | neurons | 44 | 0 | 22 | 0.01 |
| 1 | neuronal serotonin | 6 | 0 | 10 | 0.01 |
| 1 | neuronal differentiation | 2 | 0 | 2 | 0 |
| 1 | neurogenesis | 12 | 0 | 19 | 0.02 |
| 1 | nervous system | 53 | 0 | 20 | 0.02 |
| 1 | multiple sclerosis | 17 | 0 | 17 | 0.02 |
| 1 | mucosal serotonin | 2 | 0 | 5 | 0 |
| 1 | mood disorders | 2 | 0 | 1 | 0 |
| 1 | lactobacillus reuteri | 2 | 0 | 3 | 0 |
| 1 | lactobacillus plantarum ps128 | 2 | 0 | 5 | 0 |
| 1 | lactobacillus plantarum | 3 | 0 | 2 | 0 |
| 1 | intestinal barrier | 7 | 0 | 11 | 0 |
| 1 | interstitial cells | 18 | 0 | 16 | 0.02 |
| 1 | intermediate filaments | 1 | 0 | 1 | 0 |
| 1 | inflammatory response | 2 | 0 | 1 | 0 |
| 1 | inflammatory reflex | 1 | 0 | 1 | 0 |
| 1 | inflammation | 79 | 0 | 36 | 0.02 |
| 1 | infection | 7 | 0 | 10 | 0 |
| 1 | in vivo | 6 | 0 | 8 | 0 |
| 1 | immune regulation | 1 | 0 | 1 | 0 |
| 1 | health | 30 | 0 | 10 | 0 |
| 1 | gut dysbiosis | 11 | 0 | 8 | 0 |
| 1 | glial cells | 24 | 0 | 13 | 0 |
| 1 | germ free | 3 | 0 | 1 | 0 |
| 1 | gastrointestinal microflora | 1 | 0 | 1 | 0 |
| 1 | gamma aminobutyric acid | 12 | 0 | 8 | 0.01 |
| 1 | fibrillary acidic protein | 1 | 0 | 1 | 0 |
| 1 | fiber | 2 | 0 | 2 | 0 |
| 1 | expression | 47 | 0 | 13 | 0.02 |
| 1 | escherichia coli | 10 | 0 | 5 | 0 |
| 1 | enteric glia | 24 | 0 | 9 | 0.01 |
| 1 | endogenous ligands | 1 | 0 | 1 | 0 |
| 1 | endocannabinoid system | 2 | 0 | 4 | 0 |
| 1 | drinking water | 1 | 0 | 8 | 0 |
| 1 | d-lactic acid | 1 | 0 | 2 | 0 |
| 1 | disease course | 2 | 0 | 1 | 0 |
| 1 | diet | 18 | 0 | 16 | 0.02 |
| 1 | dextran sulfate | 1 | 0 | 8 | 0 |
| 1 | dendritic cells | 4 | 0 | 12 | 0.02 |
| 1 | d lactic acidosis | 1 | 0 | 2 | 0 |
| 1 | crosstalk | 1 | 0 | 2 | 0 |
| 1 | compartmentalization | 1 | 0 | 2 | 0 |
| 1 | colorectal distension | 3 | 0 | 3 | 0 |
| 1 | colitis | 7 | 0 | 28 | 0.03 |
| 1 | chronic fatigue syndrome | 1 | 0 | 2 | 0 |
| 1 | cell line | 1 | 0 | 8 | 0 |
| 1 | campylobacter jejuni | 1 | 0 | 1 | 0 |
| 1 | cajal | 5 | 0 | 5 | 0.01 |
| 1 | brain-derived neurotrophic factor | 1 | 0 | 8 | 0 |
| 1 | bowel bacterial overgrowth | 1 | 0 | 2 | 0 |
| 1 | bone formation | 1 | 0 | 2 | 0 |
| 1 | biological evolution | 1 | 0 | 5 | 0 |
| 1 | bioenergy | 1 | 0 | 6 | 0 |
| 1 | bifidobacterium longum | 3 | 0 | 25 | 0.01 |
| 1 | bifidobacterium bifidum | 1 | 0 | 4 | 0 |
| 1 | bibliometric analysis | 1 | 0 | 5 | 0 |
| 1 | behavioral symptoms | 1 | 0 | 3 | 0 |
| 1 | barrier function | 16 | 0 | 21 | 0.02 |
| 1 | bacterial infection | 2 | 0 | 6 | 0 |
| 1 | bacterial cellulose | 1 | 0 | 6 | 0 |
| 1 | ayurveda | 1 | 0 | 2 | 0 |
| 1 | auxin | 1 | 0 | 6 | 0 |
| 1 | autonomic neuropathy | 2 | 0 | 2 | 0 |
| 1 | atpase activities | 1 | 0 | 6 | 0 |
| 1 | astrocytes | 1 | 0 | 1 | 0 |
| 1 | association analysis | 1 | 0 | 3 | 0 |
| 1 | aryl hydrocarbon receptor | 9 | 0 | 12 | 0.01 |
| 1 | aromatic amino acids | 1 | 0 | 3 | 0 |
| 1 | anxiety like behavior | 24 | 5.28 | 31 | 0.04 |
| 1 | antioxidant | 1 | 0 | 6 | 0 |
| 1 | anti anxiety agents | 1 | 0 | 20 | 0 |
| 1 | animal model | 102 | 0 | 42 | 0.03 |
| 1 | amylase multigene family | 1 | 0 | 3 | 0 |
| 1 | aminobutyric acid gaba | 1 | 0 | 4 | 0 |
| 1 | amino acid transporters | 1 | 0 | 3 | 0 |
| 1 | altered expression | 1 | 0 | 7 | 0 |
| 1 | alpha 7 subunit | 1 | 0 | 1 | 0 |
| 1 | air pollution exposure | 1 | 0 | 4 | 0 |
| 1 | aganglionic bowel | 1 | 0 | 7 | 0 |
| 1 | afferent neurons | 2 | 0 | 7 | 0 |
| 1 | adeno-associated virus vector (aav) | 1 | 0 | 4 | 0 |
| 1 | acute ischemic stroke | 1 | 0 | 4 | 0 |
| 1 | activation | 20 | 0 | 36 | 0.05 |
| 1 | acid induced colitis | 1 | 0 | 5 | 0 |
| 1 | acid amide hydrolase | 1 | 0 | 5 | 0 |
| 1 | abnormality | 2 | 0 | 6 | 0 |
| 1 | abdominal surgery | 1 | 0 | 6 | 0 |
| 1 | 5 hydroxytryptamine | 4 | 0 | 12 | 0.01 |
| 1 | 5 ht4 receptors | 2 | 0 | 7 | 0 |
| 1 | 5 ht3 receptor antagonist | 1 | 0 | 5 | 0 |
| 1 | 5 ht transporter | 1 | 0 | 2 | 0 |
| 1 | 5 ht | 2 | 0 | 10 | 0 |
| 1 | 16s rrna | 1 | 0 | 4 | 0 |
| 2 | wild type | 2 | 0 | 10 | 0 |
| 2 | vitamin d | 2 | 0 | 6 | 0 |
| 2 | vancomycin | 2 | 0 | 13 | 0 |
| 2 | unclassified drug | 11 | 0 | 27 | 0.01 |
| 2 | th17 cell | 2 | 0 | 16 | 0 |
| 2 | tau protein | 2 | 0 | 1 | 0 |
| 2 | t lymphocytes | 2 | 0 | 18 | 0 |
| 2 | small intestine | 15 | 0 | 13 | 0 |
| 2 | sensitivity | 2 | 0 | 1 | 0 |
| 2 | rna sequencing | 2 | 0 | 10 | 0 |
| 2 | rna extraction | 2 | 0 | 13 | 0 |
| 2 | rna 16s | 4 | 0 | 17 | 0 |
| 2 | regulatory t lymphocyte | 2 | 0 | 18 | 0 |
| 2 | real time polymerase chain reaction | 3 | 0 | 25 | 0 |
| 2 | protein zo1 | 2 | 0 | 3 | 0 |
| 2 | protein expression | 12 | 0 | 29 | 0 |
| 2 | priority journal | 40 | 6.79 | 41 | 0.01 |
| 2 | porphyromonas gingivalis | 2 | 0 | 1 | 0 |
| 2 | phenotype | 6 | 0 | 17 | 0 |
| 2 | nitric oxide | 29 | 4.27 | 16 | 0.01 |
| 2 | nitrergic nerve cell | 2 | 0 | 19 | 0 |
| 2 | neurotransmitter agents | 2 | 0 | 9 | 0 |
| 2 | neuroglia | 2 | 0 | 8 | 0 |
| 2 | nerve cell degeneration | 2 | 0 | 5 | 0 |
| 2 | nerve cell | 14 | 0 | 30 | 0 |
| 2 | myenteric plexus | 33 | 0 | 23 | 0 |
| 2 | microbial community | 3 | 0 | 2 | 0 |
| 2 | mice | 30 | 0 | 39 | 0.01 |
| 2 | male | 17 | 0 | 59 | 0.01 |
| 2 | large intestine | 2 | 0 | 2 | 0 |
| 2 | lamina propria | 2 | 0 | 18 | 0 |
| 2 | lactobacillus rhamnosus | 2 | 0 | 3 | 0 |
| 2 | iron | 2 | 0 | 1 | 0 |
| 2 | intestine function | 2 | 0 | 15 | 0 |
| 2 | intestinal epithelial barrier | 2 | 0 | 3 | 0 |
| 2 | interleukin 6 | 3 | 0 | 21 | 0 |
| 2 | in vitro study | 4 | 0 | 29 | 0 |
| 2 | immunoreactivity | 2 | 0 | 7 | 0 |
| 2 | immunohistochemistry | 11 | 0 | 38 | 0 |
| 2 | immunocompetent cell | 3 | 0 | 22 | 0 |
| 2 | glia | 2 | 0 | 11 | 0 |
| 2 | gene expression profiling | 2 | 0 | 16 | 0 |
| 2 | gene expression | 31 | 0 | 59 | 0.03 |
| 2 | gastrointestinal transit | 7 | 0 | 22 | 0 |
| 2 | gastrointestinal tract function | 2 | 0 | 1 | 0 |
| 2 | gastrointestinal symptom | 20 | 0 | 46 | 0.03 |
| 2 | female | 11 | 0 | 49 | 0.01 |
| 2 | feces | 3 | 0 | 23 | 0.01 |
| 2 | ex vivo study | 2 | 0 | 15 | 0 |
| 2 | enzyme linked immunosorbent assay | 2 | 0 | 15 | 0 |
| 2 | drug efficacy | 14 | 0 | 40 | 0.01 |
| 2 | diverticular disease | 2 | 0 | 1 | 0 |
| 2 | disease model | 9 | 0 | 25 | 0 |
| 2 | dendritic cell | 2 | 0 | 11 | 0 |
| 2 | controlled trial | 25 | 0 | 74 | 0.02 |
| 2 | colonic motility | 11 | 0 | 17 | 0.01 |
| 2 | colon | 18 | 0 | 52 | 0.03 |
| 2 | coculture | 2 | 0 | 21 | 0 |
| 2 | cholinergic nerve cell | 7 | 0 | 42 | 0.01 |
| 2 | cell proliferation | 2 | 0 | 16 | 0 |
| 2 | cell differentiation | 2 | 0 | 21 | 0 |
| 2 | cell density | 2 | 0 | 17 | 0 |
| 2 | cell culture | 2 | 0 | 28 | 0 |
| 2 | cell contact | 2 | 0 | 21 | 0 |
| 2 | calcium cell level | 2 | 0 | 15 | 0 |
| 2 | c57bl mouse | 10 | 0 | 53 | 0.01 |
| 2 | brain injuries | 1 | 0 | 9 | 0 |
| 2 | bone marrow cell | 1 | 0 | 8 | 0 |
| 2 | biofilm | 2 | 0 | 19 | 0.01 |
| 2 | biodiversity | 1 | 0 | 14 | 0 |
| 2 | beta actin | 1 | 0 | 16 | 0 |
| 2 | autistic disorder | 1 | 0 | 10 | 0 |
| 2 | astrocyte | 1 | 0 | 9 | 0 |
| 2 | aquaporin 1 | 1 | 0 | 14 | 0 |
| 2 | anti bacterial agents | 5 | 0 | 35 | 0.01 |
| 2 | anthracene | 1 | 0 | 11 | 0 |
| 2 | animal tissue | 17 | 0 | 98 | 0.05 |
| 2 | animal cell | 11 | 0 | 48 | 0.01 |
| 2 | anatomical concepts | 1 | 0 | 14 | 0 |
| 2 | analogs and derivatives | 1 | 0 | 16 | 0 |
| 2 | amyloidosis | 1 | 0 | 20 | 0 |
| 2 | amyloid precursor protein | 1 | 0 | 20 | 0 |
| 2 | amyloid plaque | 1 | 0 | 20 | 0 |
| 2 | amyloid beta protein[1 42] | 1 | 0 | 20 | 0 |
| 2 | amyloid beta protein precursor | 1 | 0 | 20 | 0 |
| 2 | ampicillin | 2 | 0 | 22 | 0.01 |
| 2 | amine oxidase (copper containing) | 1 | 0 | 16 | 0 |
| 2 | adults | 13 | 0 | 47 | 0.02 |
| 2 | adrenergic receptors | 1 | 0 | 17 | 0 |
| 2 | adrenergic receptor stimulating agent | 1 | 0 | 17 | 0 |
| 2 | adrenergic nerve cell | 1 | 0 | 17 | 0 |
| 2 | adrenergic agents | 1 | 0 | 17 | 0 |
| 2 | acute pancreatitis | 1 | 0 | 16 | 0 |
| 2 | actinomycetales | 1 | 0 | 14 | 0 |
| 2 | acetylcholinesterase | 2 | 0 | 19 | 0 |
| 2 | 16srrna gene | 1 | 0 | 8 | 0 |
| 3 | risk | 36 | 0 | 18 | 0.01 |
| 3 | psychology | 2 | 0 | 10 | 0 |
| 3 | pregnancy | 2 | 0 | 2 | 0 |
| 3 | peristalsis | 2 | 0 | 8 | 0 |
| 3 | neurotransmission | 11 | 0 | 10 | 0 |
| 3 | microorganisms | 2 | 0 | 1 | 0 |
| 3 | mental stress | 5 | 0 | 12 | 0 |
| 3 | mental disorders | 2 | 0 | 9 | 0 |
| 3 | mental disease | 2 | 0 | 9 | 0 |
| 3 | infantile colic | 2 | 0 | 6 | 0 |
| 3 | infant | 1 | 0 | 10 | 0 |
| 3 | hypothalamus hypophysis adrenal system | 3 | 0 | 8 | 0 |
| 3 | growth | 5 | 0 | 2 | 0 |
| 3 | glucose homeostasis | 1 | 0 | 1 | 0 |
| 3 | gastrointestinal disease | 52 | 0 | 41 | 0.02 |
| 3 | gastrin | 1 | 0 | 1 | 0 |
| 3 | enteropathy | 4 | 0 | 12 | 0 |
| 3 | enteroenteric reflex | 1 | 0 | 1 | 0 |
| 3 | early life stress | 2 | 0 | 5 | 0 |
| 3 | dopaminergic neurons | 5 | 0 | 3 | 0 |
| 3 | digestive system function disorder | 5 | 0 | 17 | 0 |
| 3 | diarrhea | 6 | 0 | 21 | 0 |
| 3 | depression | 35 | 0 | 46 | 0.02 |
| 3 | deglutition disorders | 1 | 0 | 9 | 0 |
| 3 | defecation disorder | 1 | 0 | 9 | 0 |
| 3 | cytokine release | 1 | 0 | 13 | 0 |
| 3 | cyclooxygenase 2 | 2 | 0 | 13 | 0 |
| 3 | cyclic vomiting syndrome | 1 | 0 | 4 | 0 |
| 3 | corticotropin | 11 | 5.27 | 25 | 0.01 |
| 3 | controlled clinical trial | 1 | 0 | 5 | 0 |
| 3 | contractile response | 1 | 0 | 1 | 0 |
| 3 | constipation | 37 | 0 | 45 | 0.03 |
| 3 | comorbidity | 14 | 0 | 52 | 0.02 |
| 3 | commensal | 4 | 0 | 23 | 0 |
| 3 | cognition | 2 | 0 | 20 | 0 |
| 3 | trial | 1 | 0 | 13 | 0 |
| 3 | chronic gastritis | 1 | 0 | 5 | 0 |
| 3 | cholinergic receptor blocking agent | 1 | 0 | 9 | 0 |
| 3 | children | 15 | 0 | 33 | 0.02 |
| 3 | child development disorders | 1 | 0 | 16 | 0 |
| 3 | chemosensitivity | 1 | 0 | 1 | 0 |
| 3 | cell function | 1 | 0 | 2 | 0 |
| 3 | cell cycle progression | 1 | 0 | 13 | 0 |
| 3 | cell communication | 2 | 0 | 19 | 0 |
| 3 | cell activation | 2 | 0 | 21 | 0 |
| 3 | celiac disease | 4 | 0 | 5 | 0 |
| 3 | cd8 antigen | 1 | 0 | 13 | 0 |
| 3 | cd4 antigen | 1 | 0 | 13 | 0 |
| 3 | c reactive protein | 1 | 0 | 13 | 0 |
| 3 | butyrate producing bacteria | 1 | 0 | 1 | 0 |
| 3 | brain-gut-microbiota axis | 2 | 0 | 19 | 0 |
| 3 | brain-gut interaction | 1 | 0 | 1 | 0 |
| 3 | brain stem circuits | 1 | 0 | 4 | 0 |
| 3 | brain gut microbiota axis | 1 | 0 | 15 | 0 |
| 3 | brain function | 7 | 0 | 37 | 0.02 |
| 3 | brain disease | 5 | 0 | 15 | 0 |
| 3 | brain development | 5 | 0 | 36 | 0.01 |
| 3 | brain derived neurotrophic factor | 7 | 3.56 | 53 | 0.03 |
| 3 | brain | 96 | 0 | 45 | 0.02 |
| 3 | body weight | 1 | 0 | 12 | 0 |
| 3 | blood brain barrier | 17 | 0 | 26 | 0.03 |
| 3 | blautia | 1 | 0 | 6 | 0 |
| 3 | biomarkers | 1 | 0 | 6 | 0 |
| 3 | biological marker | 4 | 0 | 20 | 0.01 |
| 3 | bifidobacterium infantis 35624 | 1 | 0 | 1 | 0 |
| 3 | behavior disorder | 4 | 0 | 18 | 0 |
| 3 | bacterial translocation | 9 | 0 | 26 | 0.01 |
| 3 | bacterial overgrowth | 4 | 0 | 16 | 0 |
| 3 | bacterial commensal | 1 | 0 | 9 | 0 |
| 3 | autonomic nervous system | 14 | 0 | 54 | 0.04 |
| 3 | autism spectrum disorder | 39 | 0 | 49 | 0.03 |
| 3 | attention deficit/hyperactivity disorder | 2 | 0 | 5 | 0 |
| 3 | appetite | 2 | 0 | 17 | 0 |
| 3 | antiparkinson agents | 1 | 0 | 9 | 0 |
| 3 | antiparkinson agent | 1 | 0 | 9 | 0 |
| 3 | antidepressant agent | 2 | 0 | 12 | 0 |
| 3 | antibody | 1 | 0 | 1 | 0 |
| 3 | american migraine | 1 | 0 | 4 | 0 |
| 3 | alpha synuclein | 75 | 0 | 20 | 0.01 |
| 3 | age factors | 2 | 0 | 29 | 0.01 |
| 3 | age | 6 | 0 | 29 | 0.02 |
| 3 | affect | 2 | 0 | 29 | 0.01 |
| 3 | adverse childhood experiences | 1 | 0 | 1 | 0 |
| 3 | adolescent behavior | 1 | 0 | 17 | 0 |
| 3 | adolescent | 1 | 0 | 17 | 0 |
| 3 | acute disease | 1 | 0 | 12 | 0 |
| 3 | hydroxydopamine rat model | 1 | 0 | 2 | 0 |
| 3 | 5 acetamidosalicylic acid | 1 | 0 | 13 | 0 |
| 3 | 4 amino 5 chloro n [(hexahydro 1h pyrrolizin 1 yl)methyl] 2 methoxybenzamide | 1 | 0 | 17 | 0 |
| 4 | treatment outcome | 2 | 0 | 4 | 0 |
| 4 | therapy | 4 | 0 | 11 | 0 |
| 4 | synbiotic agent | 2 | 0 | 5 | 0 |
| 4 | superoxide dismutase | 2 | 0 | 7 | 0 |
| 4 | serotonin 4 receptor | 2 | 0 | 7 | 0 |
| 4 | review | 65 | 3.35 | 63 | 0.04 |
| 4 | quality of life | 15 | 0 | 32 | 0.01 |
| 4 | proteobacteria | 2 | 0 | 14 | 0 |
| 4 | protein aggregation | 4 | 0 | 16 | 0 |
| 4 | probiotics | 50 | 0 | 29 | 0.01 |
| 4 | prevalence | 9 | 0 | 14 | 0 |
| 4 | prebiotics | 17 | 0 | 18 | 0 |
| 4 | physiology | 57 | 4.07 | 41 | 0.01 |
| 4 | pathology | 75 | 0 | 54 | 0.02 |
| 4 | paneth cell | 2 | 0 | 8 | 0 |
| 4 | obesity | 8 | 0 | 8 | 0 |
| 4 | nonhuman | 92 | 0 | 79 | 0.03 |
| 4 | neuromuscular function | 2 | 0 | 14 | 0 |
| 4 | neurologic disease | 5 | 0 | 20 | 0 |
| 4 | neurodegenerative diseases | 17 | 0 | 16 | 0.01 |
| 4 | nerve degeneration | 4 | 0 | 8 | 0 |
| 4 | mouth | 2 | 0 | 6 | 0 |
| 4 | motor dysfunction | 2 | 0 | 17 | 0 |
| 4 | microbiota | 140 | 0 | 25 | 0.01 |
| 4 | microbial endocrinology | 5 | 0 | 2 | 0 |
| 4 | microbial dysbiosis | 2 | 0 | 2 | 0 |
| 4 | metabolism | 67 | 0 | 32 | 0.01 |
| 4 | manifestations | 2 | 0 | 5 | 0 |
| 4 | lipid metabolism | 2 | 0 | 1 | 0 |
| 4 | lactobacillus helveticus | 24 | 3.16 | 20 | 0.01 |
| 4 | ketogenic diet | 2 | 0 | 8 | 0.01 |
| 4 | irritable colon | 14 | 0 | 29 | 0.01 |
| 4 | intestine innervation | 98 | 0 | 71 | 0.02 |
| 4 | immune response | 18 | 0 | 63 | 0.04 |
| 4 | human | 105 | 0 | 86 | 0.05 |
| 4 | histone deacetylase | 2 | 0 | 7 | 0 |
| 4 | helicobacter pylori infection | 18 | 0 | 7 | 0 |
| 4 | gut motility disorder | 2 | 0 | 3 | 0 |
| 4 | gut motility | 81 | 0 | 29 | 0.02 |
| 4 | gut axis | 1 | 0 | 1 | 0 |
| 4 | gastrointestinal tract | 82 | 0 | 50 | 0.05 |
| 4 | gastrointestinal discomfort | 2 | 0 | 5 | 0 |
| 4 | fecal microbiota transplantation | 66 | 0 | 29 | 0.02 |
| 4 | fast synaptic transmission | 2 | 0 | 4 | 0 |
| 4 | extracellular matrix | 2 | 0 | 11 | 0.02 |
| 4 | etiology | 2 | 0 | 17 | 0 |
| 4 | essential oil | 2 | 0 | 12 | 0 |
| 4 | environmental factor | 3 | 0 | 5 | 0 |
| 4 | enteritis | 7 | 0 | 13 | 0 |
| 4 | enteric nervous system | 437 | 0 | 17 | 0 |
| 4 | early diagnosis | 2 | 0 | 6 | 0 |
| 4 | dysbiosis | 35 | 0 | 67 | 0.03 |
| 4 | drug therapy | 7 | 0 | 21 | 0 |
| 4 | digestive system | 3 | 0 | 9 | 0 |
| 4 | degenerative disease | 4 | 0 | 13 | 0 |
| 4 | corticotropin releasing hormone | 1 | 0 | 1 | 0 |
| 4 | clinical feature | 1 | 0 | 5 | 0 |
| 4 | central nervous system | 55 | 0 | 42 | 0.03 |
| 4 | broiler chickens | 1 | 0 | 3 | 0 |
| 4 | breath analysis | 1 | 0 | 6 | 0 |
| 4 | biomedical research | 1 | 0 | 10 | 0 |
| 4 | biological model | 2 | 0 | 11 | 0 |
| 4 | biodegradation | 1 | 0 | 2 | 0 |
| 4 | bifidobacterium longum r0175 | 3 | 0 | 1 | 0 |
| 4 | bifidobacterium | 8 | 0 | 60 | 0.03 |
| 4 | betacoronavirus | 1 | 0 | 19 | 0 |
| 4 | bacteroidetes | 3 | 0 | 31 | 0.01 |
| 4 | bacteriophage | 1 | 0 | 14 | 0 |
| 4 | bacterial growth | 1 | 0 | 16 | 0 |
| 4 | axonal transport | 1 | 0 | 2 | 0 |
| 4 | antimicrobial activity | 1 | 0 | 4 | 0 |
| 4 | analysis reveals | 1 | 0 | 4 | 0 |
| 4 | amyotrophic lateral sclerosis (als) | 1 | 0 | 5 | 0 |
| 4 | amyotrophic lateral sclerosis | 7 | 0 | 29 | 0.02 |
| 4 | adverse drug reaction | 1 | 0 | 10 | 0 |
| 4 | acute tryptophan depletion | 1 | 0 | 4 | 0 |
| 4 | acute gastroenteritis | 1 | 0 | 6 | 0 |
| 4 | actinobacteria | 1 | 0 | 10 | 0 |
| 4 | abdominal pain | 18 | 0 | 64 | 0.05 |
| 4 | abdominal distension | 1 | 0 | 15 | 0 |
| 4 | 5 ht3 receptors | 1 | 0 | 4 | 0 |
| 5 | visceral pain | 6 | 0 | 6 | 0 |
| 5 | tumor necrosis factor | 8 | 0 | 22 | 0 |
| 5 | tryptophan hydroxylase 1 | 2 | 0 | 5 | 0 |
| 5 | substance P | 5 | 0 | 2 | 0 |
| 5 | structural changes | 2 | 0 | 1 | 0 |
| 5 | spinal ganglion | 3 | 0 | 8 | 0 |
| 5 | spinal cord | 2 | 0 | 2 | 0 |
| 5 | regulatory mechanism | 2 | 0 | 6 | 0 |
| 5 | parasympathetic nerve | 2 | 0 | 6 | 0 |
| 5 | neuropeptide | 2 | 0 | 10 | 0 |
| 5 | neurological disorders | 8 | 0 | 16 | 0.03 |
| 5 | neuroimmunomodulation | 2 | 0 | 5 | 0 |
| 5 | nervous system function | 2 | 0 | 10 | 0 |
| 5 | mucosal immunity | 6 | 0 | 6 | 0 |
| 5 | motoneuron | 3 | 0 | 3 | 0 |
| 5 | microbial metabolites | 7 | 0 | 4 | 0.01 |
| 5 | lymphoid cell | 2 | 0 | 3 | 0 |
| 5 | leptin | 2 | 0 | 5 | 0 |
| 5 | intestine epithelium | 2 | 0 | 7 | 0 |
| 5 | intestinal permeability | 33 | 0 | 19 | 0.01 |
| 5 | interleukin 1beta | 4 | 0 | 8 | 0 |
| 5 | interleukin 10 | 3 | 0 | 14 | 0 |
| 5 | immunomodulation | 2 | 0 | 5 | 0 |
| 5 | hypothalamus hypophysis system | 2 | 0 | 6 | 0 |
| 5 | homeostasis | 14 | 0 | 24 | 0.01 |
| 5 | histamine | 3 | 0 | 11 | 0 |
| 5 | gut microbiota-brain axis | 2 | 0 | 6 | 0 |
| 5 | gastrointestinal hormone | 2 | 0 | 5 | 0 |
| 5 | gastrointestinal absorption | 2 | 0 | 8 | 0 |
| 5 | firmicutes | 2 | 0 | 6 | 0 |
| 5 | disease predisposition | 2 | 0 | 7 | 0 |
| 5 | diagnosis | 4 | 0 | 2 | 0 |
| 5 | defecation | 2 | 0 | 17 | 0 |
| 5 | coronavirus disease 2019 | 2 | 0 | 4 | 0 |
| 5 | calcitonin gene related peptide | 3 | 0 | 16 | 0 |
| 5 | brain stem | 2 | 0 | 7 | 0 |
| 5 | blood flow | 1 | 0 | 3 | 0 |
| 5 | bacteroides | 1 | 0 | 8 | 0 |
| 5 | bacterial microbiome | 1 | 0 | 4 | 0 |
| 5 | bacterial metabolism | 1 | 0 | 4 | 0 |
| 5 | autoimmune disease | 1 | 0 | 12 | 0 |
| 5 | antigen presenting cell | 1 | 0 | 12 | 0 |
| 5 | anorectal pressure | 1 | 0 | 9 | 0 |
| 5 | anatomy and histology | 1 | 0 | 8 | 0 |
| 5 | anatomy | 2 | 0 | 16 | 0 |
| 5 | anal canal | 1 | 0 | 9 | 0 |
| 5 | amygdala | 1 | 0 | 11 | 0 |
| 5 | aminoglycoside antibiotics | 1 | 0 | 1 | 0 |
| 5 | alzheimer’s disease | 1 | 0 | 1 | 0 |
| 5 | allergic reaction | 1 | 0 | 4 | 0 |
| 5 | akkermansia | 2 | 0 | 16 | 0 |
| 5 | aging | 2 | 0 | 21 | 0.01 |
| 5 | agents interacting with transmitter | 5 | 0 | 45 | 0.02 |
| 5 | adrenergic system | 3 | 0 | 15 | 0 |
| 5 | adenosine triphosphate | 2 | 0 | 12 | 0 |
| 5 | acetylcholine | 2 | 0 | 16 | 0 |
| 5 | absorption | 3 | 0 | 11 | 0 |
| 5 | 4 aminobutyric acid a receptor | 2 | 0 | 25 | 0.01 |
| 5 | 4 aminobutyric acid | 3 | 0 | 25 | 0.01 |
| 6 | transforming growth factor (tgf)-β | 1 | 0 | 2 | 0 |
| 6 | smad signaling | 1 | 0 | 2 | 0 |
| 6 | receptor cross talk | 1 | 0 | 2 | 0 |
| 6 | pattern recognition receptor signaling | 1 | 0 | 2 | 0 |
| 6 | mucin | 1 | 0 | 4 | 0 |
| 6 | mitogen activated protein kinase | 1 | 0 | 2 | 0 |
| 6 | lotus | 1 | 0 | 4 | 0 |
| 6 | intestine injury | 1 | 0 | 5 | 0 |
| 6 | intestine epithelium cell | 1 | 0 | 3 | 0 |
| 6 | intestinal epithelial cells | 5 | 0 | 4 | 0 |
| 6 | interferon regulatory factor | 1 | 0 | 3 | 0 |
| 6 | innate immunity | 11 | 3.26 | 14 | 0 |
| 6 | immunopathology | 1 | 0 | 3 | 0 |
| 6 | immunoglobulin enhancer binding protein | 1 | 0 | 3 | 0 |
| 6 | immunoglobulin a | 1 | 0 | 5 | 0 |
| 6 | hyperalgesia | 1 | 0 | 5 | 0 |
| 6 | host pathogen interaction | 6 | 0 | 10 | 0 |
| 6 | host | 4 | 0 | 5 | 0 |
| 6 | hippocampus | 1 | 0 | 5 | 0 |
| 6 | goblet cell | 1 | 0 | 5 | 0 |
| 6 | germfree animal | 1 | 0 | 5 | 0 |
| 6 | gamma interferon | 1 | 0 | 5 | 0 |
| 6 | exploratory behavior | 1 | 0 | 5 | 0 |
| 6 | enzyme activity | 2 | 0 | 10 | 0 |
| 6 | enterocolitis | 2 | 0 | 1 | 0 |
| 6 | enterococcus | 1 | 0 | 1 | 0 |
| 6 | enterobacteriaceae | 1 | 0 | 3 | 0 |
| 6 | endocrine system | 1 | 0 | 5 | 0 |
| 6 | drug mechanism | 2 | 0 | 8 | 0 |
| 6 | drug formulation | 1 | 0 | 5 | 0 |
| 6 | disease duration | 1 | 0 | 3 | 0 |
| 6 | disease association | 1 | 0 | 6 | 0 |
| 6 | diet supplementation | 7 | 0 | 23 | 0.02 |
| 6 | defensin | 2 | 0 | 7 | 0 |
| 6 | defecation habit | 1 | 0 | 5 | 0 |
| 6 | cytokine production | 1 | 0 | 5 | 0 |
| 6 | corpus striatum | 1 | 0 | 5 | 0 |
| 6 | colic | 1 | 0 | 1 | 0 |
| 6 | clostridium tetani | 1 | 0 | 5 | 0 |
| 6 | citrobacter | 1 | 0 | 1 | 0 |
| 6 | chronic inflammation | 2 | 0 | 31 | 0.01 |
| 6 | chronic disease | 2 | 0 | 42 | 0.01 |
| 6 | central nervous system function | 1 | 0 | 6 | 0 |
| 6 | catecholamine | 3 | 0 | 1 | 0 |
| 6 | carbohydrate | 1 | 0 | 5 | 0 |
| 6 | behavior modification | 1 | 0 | 7 | 0 |
| 6 | bacterial strain | 3 | 0 | 30 | 0.02 |
| 6 | bacterial colonization | 2 | 0 | 30 | 0.01 |
| 6 | bacitracin | 1 | 0 | 5 | 0 |
| 6 | aquatic environment | 1 | 0 | 7 | 0 |
| 6 | animalia | 1 | 0 | 7 | 0 |
| 6 | animal behavior | 5 | 0 | 50 | 0.03 |
| 6 | animal | 83 | 0 | 145 | 0.16 |
| 6 | analgesia | 2 | 0 | 27 | 0.01 |
| 6 | amygdaloid nucleus | 1 | 0 | 29 | 0 |
| 6 | alteration of host behaviour | 1 | 0 | 7 | 0 |
| 6 | allergic encephalomyelitis | 1 | 0 | 29 | 0 |
| 6 | abdominal discomfort | 1 | 0 | 29 | 0 |
| 7 | ulcerative colitis | 27 | 0 | 14 | 0.01 |
| 7 | trimethylamine n oxide | 2 | 0 | 1 | 0 |
| 7 | sympathetic nerve | 2 | 0 | 9 | 0 |
| 7 | supplementation | 2 | 0 | 2 | 0 |
| 7 | submucosal plexus | 8 | 0 | 14 | 0 |
| 7 | shannon index | 2 | 0 | 2 | 0 |
| 7 | regulatory t cells | 6 | 0 | 5 | 0 |
| 7 | protein coupled receptor | 2 | 0 | 2 | 0 |
| 7 | protease activated receptors | 2 | 0 | 9 | 0 |
| 7 | primary afferent nerves | 2 | 0 | 9 | 0 |
| 7 | nerve stimulation protects | 2 | 0 | 9 | 0 |
| 7 | myenteric neurons | 16 | 0 | 24 | 0.02 |
| 7 | mental health | 6 | 0 | 6 | 0 |
| 7 | mediterranean diet | 2 | 0 | 1 | 0 |
| 7 | intestinal barrier function | 4 | 0 | 9 | 0 |
| 7 | international scientific association | 2 | 0 | 2 | 0 |
| 7 | induced obesity | 2 | 0 | 3 | 0 |
| 7 | induced mucin secretion | 2 | 0 | 9 | 0 |
| 7 | immunoglobulin a secretion | 2 | 0 | 9 | 0 |
| 7 | human colonic microbiota | 1 | 0 | 2 | 0 |
| 7 | glucose metabolism | 4 | 0 | 6 | 0 |
| 7 | glucagon like peptide 1 | 23 | 3.29 | 24 | 0.03 |
| 7 | gastric bypass surgery | 2 | 0 | 4 | 0 |
| 7 | epithelium cell | 11 | 0 | 26 | 0.02 |
| 7 | epithelial barrier function | 2 | 0 | 2 | 0 |
| 7 | enteroendocrine cell | 40 | 0 | 22 | 0.02 |
| 7 | diet induced obesity | 6 | 0 | 3 | 0 |
| 7 | dependent insulinotropic polypeptide | 2 | 0 | 4 | 0 |
| 7 | cryptome proteins | 1 | 0 | 8 | 0 |
| 7 | crohn disease | 16 | 0 | 18 | 0.02 |
| 7 | conductance | 1 | 0 | 2 | 0 |
| 7 | comprehensive proteomic analysis | 1 | 0 | 8 | 0 |
| 7 | classification of bioactive peptides | 1 | 0 | 8 | 0 |
| 7 | circadian clock | 2 | 0 | 6 | 0 |
| 7 | chain fatty acids | 128 | 7.33 | 10 | 0.01 |
| 7 | cancer | 4 | 0 | 1 | 0 |
| 7 | body mass index | 2 | 0 | 4 | 0 |
| 7 | bioactive peptides | 2 | 0 | 13 | 0.03 |
| 7 | bile acid | 21 | 0 | 14 | 0.02 |
| 7 | bidirectional aggregation | 1 | 0 | 7 | 0 |
| 7 | beta phenylethylamine | 1 | 0 | 5 | 0 |
| 7 | beta lactoglobulin | 1 | 0 | 8 | 0 |
| 7 | availability | 1 | 0 | 2 | 0 |
| 7 | anxiety stress scales | 1 | 0 | 1 | 0 |
| 7 | antioxidant peptides | 1 | 0 | 8 | 0 |
| 7 | antinutritional factors | 1 | 0 | 8 | 0 |
| 7 | antihypertensive peptides | 1 | 0 | 8 | 0 |
| 7 | amino acids | 5 | 0 | 11 | 0 |
| 7 | american association | 1 | 0 | 2 | 0 |
| 7 | adjunctive therapy | 1 | 0 | 6 | 0 |
| 7 | adipose | 3 | 0 | 5 | 0 |
| 7 | adenylate cyclase | 1 | 0 | 2 | 0 |
| 7 | active ulcerative colitis | 1 | 0 | 6 | 0 |
| 7 | acid receptor tgr5 | 1 | 0 | 2 | 0 |
| 7 | acetylcholine receptors | 1 | 0 | 5 | 0 |
| 7 | 16s ribosomal rna | 1 | 0 | 2 | 0 |
| 8 | signal transduction | 17 | 0 | 21 | 0 |
| 8 | nervous system inflammation | 4 | 0 | 5 | 0 |
| 8 | natural killer cell | 1 | 0 | 3 | 0 |
| 8 | muscle | 2 | 0 | 3 | 0 |
| 8 | monocyte chemotactic protein 1 | 1 | 0 | 3 | 0 |
| 8 | monocyte | 1 | 0 | 3 | 0 |
| 8 | mesalazine | 1 | 0 | 3 | 0 |
| 8 | mast cell | 18 | 0 | 25 | 0.01 |
| 8 | macrophages | 3 | 0 | 13 | 0 |
| 8 | intestinal mucosa | 14 | 0 | 32 | 0.01 |
| 8 | interleukin 8 | 1 | 0 | 3 | 0 |
| 8 | innervation | 20 | 0 | 31 | 0.01 |
| 8 | immunoregulation | 2 | 0 | 2 | 0 |
| 8 | immunopathogenesis | 1 | 0 | 3 | 0 |
| 8 | immunity | 33 | 5.75 | 60 | 0.03 |
| 8 | hypersensitivity | 2 | 0 | 9 | 0 |
| 8 | gut-brain axis | 148 | 0 | 16 | 0.01 |
| 8 | gut microbiota | 469 | 0 | 11 | 0 |
| 8 | gut | 53 | 0 | 90 | 0.09 |
| 8 | gastrointestinal mucosa | 1 | 0 | 4 | 0 |
| 8 | gastroenteritis | 1 | 0 | 4 | 0 |
| 8 | gamma interferon inducible protein | 1 | 0 | 4 | 0 |
| 8 | food allergy | 3 | 0 | 9 | 0 |
| 8 | eosinophil | 1 | 0 | 4 | 0 |
| 8 | disease progression | 1 | 0 | 4 | 0 |
| 8 | dietary restriction | 1 | 0 | 4 | 0 |
| 8 | cytotoxic t lymphocyte | 1 | 0 | 4 | 0 |
| 8 | cytology | 3 | 0 | 14 | 0 |
| 8 | cytokines | 1 | 0 | 10 | 0 |
| 8 | cytokine | 3 | 0 | 17 | 0 |
| 8 | cyclic amp dependent protein kinase | 1 | 0 | 10 | 0 |
| 8 | confocal microscopy | 1 | 0 | 10 | 0 |
| 8 | colon mucosa | 1 | 0 | 10 | 0 |
| 8 | clinical trial | 3 | 0 | 7 | 0 |
| 8 | cholecystokinin | 3 | 0 | 10 | 0 |
| 8 | cell senescence | 1 | 0 | 4 | 0 |
| 8 | cell interaction | 1 | 0 | 10 | 0 |
| 8 | cell infiltration | 2 | 0 | 15 | 0 |
| 8 | cd8+ t lymphocyte | 1 | 0 | 4 | 0 |
| 8 | cd4+ t lymphocyte | 1 | 0 | 4 | 0 |
| 8 | caloric restriction | 2 | 0 | 14 | 0.01 |
| 8 | caecal patch | 1 | 0 | 2 | 0 |
| 8 | blood | 1 | 0 | 4 | 0 |
| 8 | b lymphocyte | 1 | 0 | 4 | 0 |
| 8 | autacoid | 2 | 0 | 19 | 0 |
| 8 | auerbach plexus | 1 | 0 | 4 | 0 |
| 8 | atypical antipsychotics | 1 | 0 | 3 | 0 |
| 8 | artificial milk | 1 | 0 | 3 | 0 |
| 8 | antipsychotic-induced weight gain | 1 | 0 | 3 | 0 |
| 8 | antiinflammatory agent | 6 | 0 | 52 | 0.02 |
| 8 | antibody production | 1 | 0 | 38 | 0.01 |
| 8 | antibiotics | 10 | 0 | 74 | 0.06 |
| 8 | anti inflammatory agents | 1 | 0 | 10 | 0 |
| 8 | anatomical evidence | 2 | 0 | 4 | 0 |
| 8 | aganglionosis | 1 | 0 | 2 | 0 |
| 8 | adaptive immunity | 6 | 0 | 78 | 0.05 |
| 8 | 5 hydroxyindoleacetic acid | 1 | 0 | 7 | 0 |

**2. Supplementary figures**

**Supplementary Figure 1** Analysis of collaborative network visualization of countries/regions in CiteSpace. The size of nodes represents the co-occurrence frequency. Red nodes indicate countries/regions with high citation bursts, while nodes with a purple ring represent higher centrality.

**Supplementary Figure 2** Analysis of collaborative network visualization of institutions in CiteSpace. The size of nodes represents the co-occurrence frequency. Red nodes indicate countries/regions with high citation bursts, while nodes with a purple ring represent higher centrality.

**Supplementary Figure 3** The top 15 authors with the strongest citation bursts.

**Supplementary Figure 4** The top 18 keywords with the strongest citation bursts.
